# Pre-existing chromatin accessibility of switchable repressive compartment delineates cell plasticity

**DOI:** 10.1093/nsr/nwab230

**Published:** 2021-12-31

**Authors:** Xiaolong Ma, Xuan Cao, Linying Zhu, Ying Li, Xuelong Wang, Baihua Wu, Gang Wei, Lijian Hui

**Affiliations:** State Key Laboratory of Cell Biology, Shanghai Institute of Biochemistry and Cell Biology, Center for Excellence in Molecular Cell Science, Chinese Academy of Sciences; University of Chinese Academy of Sciences, Shanghai 200031, China; CAS Key Laboratory of Computational Biology, Shanghai Institute of Nutrition and Health, Chinese Academy of Sciences, University of Chinese Academy of Sciences, Shanghai 200031, China; State Key Laboratory of Cell Biology, Shanghai Institute of Biochemistry and Cell Biology, Center for Excellence in Molecular Cell Science, Chinese Academy of Sciences; University of Chinese Academy of Sciences, Shanghai 200031, China; CAS Key Laboratory of Computational Biology, Shanghai Institute of Nutrition and Health, Chinese Academy of Sciences, University of Chinese Academy of Sciences, Shanghai 200031, China; CAS Key Laboratory of Computational Biology, Shanghai Institute of Nutrition and Health, Chinese Academy of Sciences, University of Chinese Academy of Sciences, Shanghai 200031, China; State Key Laboratory of Cell Biology, Shanghai Institute of Biochemistry and Cell Biology, Center for Excellence in Molecular Cell Science, Chinese Academy of Sciences; University of Chinese Academy of Sciences, Shanghai 200031, China; CAS Key Laboratory of Computational Biology, Shanghai Institute of Nutrition and Health, Chinese Academy of Sciences, University of Chinese Academy of Sciences, Shanghai 200031, China; State Key Laboratory of Cell Biology, Shanghai Institute of Biochemistry and Cell Biology, Center for Excellence in Molecular Cell Science, Chinese Academy of Sciences; University of Chinese Academy of Sciences, Shanghai 200031, China; Institute for Stem Cell and Regeneration, Chinese Academy of Sciences, Beijing 100101, China; Bio-Research Innovation Center, Shanghai Institute of Biochemistry and Cell Biology, Suzhou 215121, China; School of Life Science and Technology, ShanghaiTech University, Shanghai 201210, China; School of Life Science, Hangzhou Institute for Advanced Study, University of Chinese Academy of Sciences, Hangzhou 310024, China

**Keywords:** cell plasticity, intrinsic properties, compartment-switchable regions, mosaic status, pre-existing accessibility, hepatic transdifferentiation

## Abstract

Cell plasticity endows differentiated cells with competence to be reprogrammed to other lineages. Although extrinsic factors driving cell-identity conversion have been extensively characterized, it remains elusive which intrinsic epigenetic attributes, including high-order chromatin organization, delineate cell plasticity. By analysing the transcription-factor-induced transdifferentiation from fibroblasts to hepatocytes, we uncovered contiguous compartment-switchable regions (CSRs) as a unique chromatin unit. Specifically, compartment B-to-A CSRs, enriched with hepatic genes, possessed a mosaic status of inactive chromatin and pre-existing and continuous accessibility in fibroblasts. Pre-existing accessibility enhanced the binding of inducible factor Foxa3, which triggered epigenetic activation and chromatin interaction as well as hepatic gene expression. Notably, these changes were restrained within B-to-A CSR boundaries that were defined by CTCF occupancy. Moreover, such chromatin organization and mosaic status were detectable in different cell types and involved in multiple reprogramming processes, suggesting an intrinsic chromatin attribute in understanding cell plasticity.

## INTRODUCTION

Numerous lines of evidence have demonstrated that differentiated cells could be reprogrammed into other identities, mainly through dedifferentiation and transdifferentiation [1–3]. This feature of differentiated cells is termed as cell plasticity, which is fundamental for regenerative medicine. During the establishment of new cell identity, extrinsic factors, e.g. transcription factors and external compounds, trigger remarkable epigenetic reprogramming of original cells [4,5]. While many studies have characterized reprogramming in this direction, there is limited understanding about the intrinsic attribute underlying cell plasticity in response to extrinsic factors [6,7].

During cell-identity conversion, differentiated cells undergo remarkable epigenetic reprogramming to activate genes that are related to the new identity [4,5]. The induction of new cell-identity-related genes is largely associated with changes in histone modifications, chromatin accessibility and high-order chromatin structures [3–5]. For example, an increase in active histone markers H3K4me1 and H3K27ac and a decrease in repressive marker H3K27me3 were found at pluripotency genes during the dedifferentiation of fibroblasts to induced pluripotent stem cells (iPSCs) *in vitro* [8–10]. In addition, it was lately reported that hepatocytes and intestine enteroendocrine cells could be dedifferentiated to progenitors *in vivo* to regenerate injured tissues [11,12]. Interestingly, it was found that chromatin accessibility in genes related to progenitors remained permissive in these differentiated cells prior to injury [13–15], which functioned as an intrinsic attribute endowing cell plasticity *in vivo* in responding to injury-induced dedifferentiation.

Recent studies have also shown that 3D chromatin structures, such as compartments, topologically associating domains (TADs) and loops [16–18], are actively involved in transcriptional regulation [5,19]. Compartments are mega-base units and segregate genomes into A and B types, associated with active and inactive chromatin status, respectively [16]. Within compartments, TADs and loops are found to possess strong insulation to distinguish themselves from flanking regions [18–22]. Intriguingly, whereas the spatial position of TADs and loops are largely conserved across different cell types, chromatin compartments show remarkable cell-type specificity [23,24], therefore underlining an association between compartmentalization and cell-identity conversion.

Indeed, compartment switch was associated with the formation of new cell identities [25]. Although compartment switch in transdifferentiation has not yet been characterized, pioneer studies on dedifferentiation have provided an outline of the kinetics of chromatin compartmentalization. For example, switch of compartment B to A was reported to accompany the establishment of active chromatin during the formation of iPSCs [26,27]. However, it remains uncharacterized whether chromatin compartmentalization, together with other chromatin statuses, constitutes any intrinsic attributes underlying cell plasticity in responding to reprogramming factors. Notably, it was recently uncovered that compartments A and B were divided into subtypes according to distinct patterns of chromatin statuses [18], suggesting that compartments were not homogeneous structures. It also highlights a possibility that different compartment regions may possess unique features accounting for their responding to reprogramming factors.

Here, using Foxa3-, Hnf1a- and Gata4 (3TF)-induced hepatic transdifferentiation [28], we characterized chromatin changes during conversion from fibroblasts to hepatocytes. We uncovered compartment-switchable regions (CSRs) that showed contiguous local compartment switch. Specifically, compartment B-to-A CSRs were featured with a mosaic status in fibroblasts, showing pre-existing and continuous accessibility in a repressive compartment. Such pre-existing accessibility increased the binding of Foxa3, followed by compartment switch, the addition of active histone modifications and hepatocyte-specific gene expression. Notably, the chimeric feature was identified to be involved in multiple types of conversions. Together, our data proposed that pre-existing chromatin accessibility in B-to-A CSRs could be served as a framework to understanding the intrinsic molecular attributes of cell plasticity.

## RESULTS

### CSRs as the unit in establishing hepatocyte-like chromatin structures

**Figure 1.**
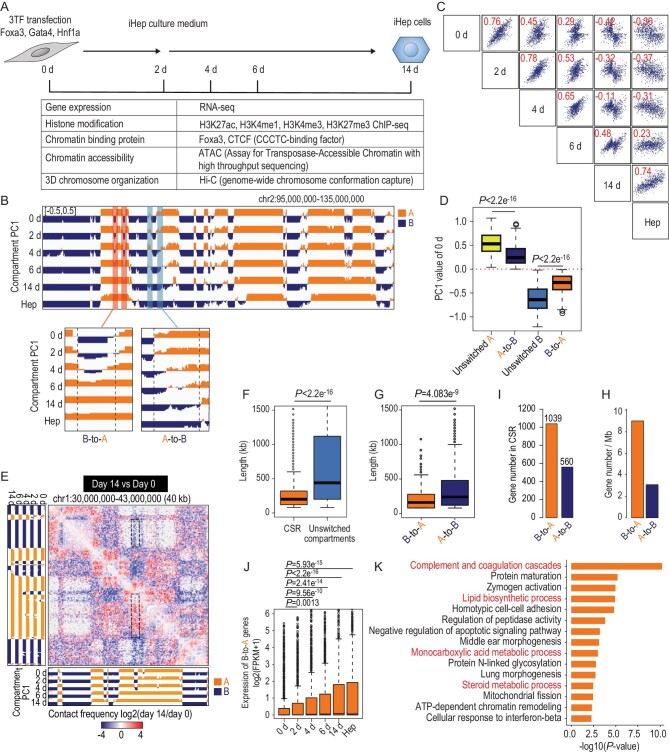
Establishment of hepatocyte-like chromatin structures. (A) Schematic overview of the experimental design. (B) A representative example of the dynamic compartmentalization during hepatic conversion. Compartment is shown by PC1 track at 40-kb resolution. Red shadows and blue shadows highlight regions with B-to-A and A-to-B compartment changes, respectively. (C) Scatter plot of average PC1 values for switched compartment regions between different time points during the hepatic conversion. Pearson correlation coefficients are shown in red, from –0.30 to 0.74. (D) Box plots of PC1 values of unswitched compartment A, B and A-to-B, B-to-A CSRs. Wilcoxon rank sum test was applied. (E) Differential Hi-C heat map showing altercation of chromatin interaction change of fibroblasts and iHeps at Day 14 in chr1:30 000 000–43 000 000 at 40-kb resolution, which was determined by subtraction of the contact matrix of fibroblasts and iHep cells at Day 14. Dashed box highlights the interaction changes between compartment-switchable regions and unswitched compartments. (F) Box plots of genomic length of CSRs and unswitched compartments during hepatic conversion. The thick line in the box indicates the median value. The whiskers out of the box indicate the 1.5-fold of the interquartile range. Wilcoxon rank sum test was applied. (G) Box plots of the genomic length of B-to-A CSRs and A-to-B CSRs. Wilcoxon rank sum test was applied. (H) Average gene density of B-to-A CSRs and A-to-B CSRs. (I) Numbers of genes located in B-to-A CSRs and A-to-B CSRs. (J) Box plots of expression levels of genes located in B-to-A CSRs. Wilcoxon rank sum test was applied. (K) Gene-ontology enrichment analysis for upregulated genes in B-to-A CSRs using Metascape. Pathways related to hepatic functions are highlight in red.

Mouse-tail-tip fibroblasts were converted into functional hepatocyte-like cells (iHep) by the transduction of Foxa3, Gata4 and Hnf1a (3TFs) [28] efficiently using an optimized protocol (see the ‘Materials and methods’ section). Hepatic phenotypes were stably established at Day 14 and maintained thereafter. Upon 3TF induction, 89.5% of cells co-expressed all 3TFs at Day 2 (Fig. S1A). At Day 14, most of the cells showed epithelial morphology using E-cadherin staining (Fig. S1B) and 92.4% of the cells expressed mature hepatocyte transcription factor Hnf4a (Fig. S1C). These data showed highly efficient hepatic conversion as previously reported [29]. Gene-expression profiles showed a trajectory from fibroblasts to hepatocytes using principal component analysis (Fig. S2A). Differentially expressed genes (DEGs) related to hepatic functions were induced, including those involved in metabolic processes (Fig. S2B), whereas DEGs highly expressed in fibroblasts were remarkably downregulated (Fig. S2C). Moreover, normalized histone modifications and chromatin accessibility showed highly dynamic changes during hepatic conversion and trajectories of these changes supported the conversion from fibroblasts to hepatocytes (Figs 1A and S2D). When induced hepatic genes were specifically analysed, we found that overall chromatin accessibility and H3K27ac modification were increased and H3K27me3 levels were reduced (Fig. S2E–G).

Chromatin structure was determined by the high-throughput chromosome conformation capture assay (Hi-C) (Table S1). Correlation and trajectory analyses of compartmentalization changes showed a shift from fibroblasts to hepatocytes (Fig. S3A). The proportion of genome that switched between compartment A and B was gradually increased to 13.7% during hepatic conversion (Fig. S3B). On the other hand, among regions showing different compartmentalization between fibroblasts and hepatocytes, 8.4% of genome regions remained unswitched in iHep cells (2.0% in compartment A and 6.4% in compartment B). Interestingly, genome regions with compartment switch showed concordant changes of PC1 in contiguous bins (Fig. 1B). When compared with hepatocytes, a change from negative correlation to positive correlation was observed in these regions along the hepatic conversion (Fig. 1C).

Contiguous bins with the same trend of PC1 changes during hepatic conversion were merged as a unit and termed as CSRs (Fig. 1B). In total, 1235 CSRs were identified, with 975 CSRs showing changes either from compartment B to A (B-to-A CSRs, 478 CSRs, 38.7%) or from A to B (A-to-B CSRs, 497 CSRs, 40.2%) (Figs 1B, and S3D and E). Notably, 77.1% of these switched regions were overlapped with chromatin showing different compartments between fibroblasts and hepatocytes (Fig. S3C). These overlapped regions accounted for 52.2% of the total number of different compartments between fibroblasts and hepatocytes (Fig. S3C). When compared with unswitched compartment A and B regions, CSRs showed smaller absolute PC1 values at Day 0 (Fig. 1D). In addition to PC1 values, chromatin interactions related to CSRs were also changed remarkably (Figs 1E, and S3F and G). Specifically, B-to-A CSRs preferentially interacted with other compartment B regions at Day 0 and these interactions dramatically decreased at Day 14. Most CSRs only occupied a part of the compartment in which they were originally located. Consequently, CSRs showed the median size of 200 kb—much shorter than the median size (560 kb) of unswitched compartments (Fig. 1F). B-to-A CSRs (160 kb) were slightly shorter than A-to-B CSRs (240 kb) (Fig. 1G).

Overall, 1039 genes were in B-to-A CSRs with a density of 8.5 genes per Mb (Fig. 1H), whereas there were 560 genes located in A-to-B CSRs (3 genes per Mb) (Fig. 1I). Significantly increased expression was also found for genes in B-to-A CSRs (Fig. 1J). In total, there were 190 DEGs induced in B-to-A CSRs (Fig. S4A). Gene-ontology analysis validated that these genes were enriched in multiple hepatic functions, including complement and coagulation cascades, steroid metabolic process, the lipid biosynthetic process and the monocarboxylic acid metabolic process (Fig. 1K). Specifically, genes important in hepatic functions were identified, including secretory protein genes *Albumin* and *Cp*; cytochrome P450 enzyme genes *Cyp2d22* and *Cyp3a13*; complement and coagulation cascades genes *C3*, *Fga* and *Fgb*; and regulatory genes associated with hepatic identity *Igf2* and *Insig1*. The expression of genes in A-to-B CSRs was decreased during hepatic conversion (Fig. S4B). Interestingly, fibroblast-related pathways were not enriched in these genes (Fig. S4C and D). These findings suggested that B-to-A CSRs might be an important chromatin unit in hepatic identity establishment.

The changes of TADs and loop interaction were also analysed. On average, 3422 TADs were captured at each time point by Topdom (see the ‘Materials and methods’ section), with an average length of 678 kb that was much longer than CSRs (Fig. S5A). In line with previous findings [17,23], positioning of TADs remained stable during hepatic conversion (Fig. S5B). A total of 9343 loops were identified with median length of 200 kb (Fig. S5A)

and loop anchors were validated by the occupancy of CTCF (8737 of 13 735 loop anchors, Fig. S5C). Interestingly, only a small fraction of the loops showed significantly altered chromatin interactions (547 increased and 273 reduced) (Fig. S5D). We identified 106 upregulated DEGs and 83 downregulated DEGs located within loops showing increased interaction. In addition, 9 upregulated DEGs and 26 downregulated DEGs were found in loops showing decreased interaction.

### Pre-existing accessibility depicts a mosaic feature of B-to-A CSRs in fibroblasts

**Figure 2.**
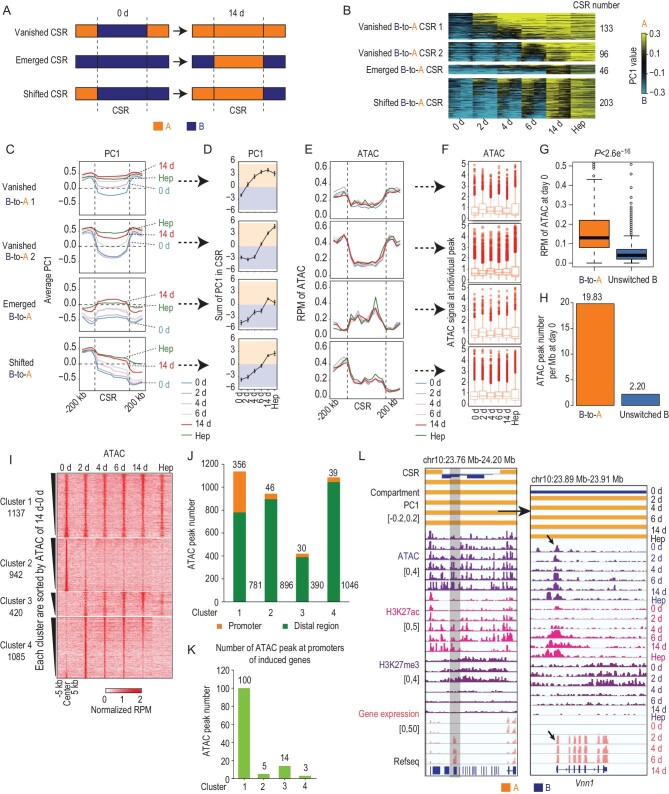
Pre-existing chromatin accessibility depicts the mosaic feature in B-to-A CSRs. (A) Three patterns of the compartment switch in B-to-A CSRs. (B) Heat maps showing four groups of PC1 values in B-to-A CSRs after k-means clustering, at 40-kb resolution. All shifted CSRs were aligned as unswitched A_CSR_unswitched B in the analysis. Regions shown in the heat map are CSRs. (C) Kinetics of compartmentalization corresponding to B-to-A CSRs and flanking regions. PC1 value was used to profile compartmentalization. (D) Broken line graph of the kinetics of PC1 values in four groups of B-to-A CSRs. Only the PC1 values within the CSRs were summed and data are presented as mean ± s.d. (E) Distribution of chromatin accessibility at four groups of B-to-A CSRs and flanking regions. (F) Box plots showing the kinetics of chromatin accessibility at individual ATAC peaks in four groups of B-to-A CSRs. (G) Box plots of average ATAC signals in B-to-A CSRs and unswitched compartment B regions at Day 0. Wilcoxon rank sum test was applied. (H) Average numbers of ATAC peaks in B-to-A CSRs and unswitched compartment B regions at Day 0. (I) Heat maps showing the signal of four clusters of ATAC peaks. (J and K) Number of ATAC peaks associated with gene promoters (top, orange) and distal regions (bottom, green) (J), and induced genes (K). (L) Representative example of a B-to-A CSR where hepatic gene *Vnn1* is located.

According to compartment-switch patterns, CSRs were classified into three groups: a whole compartment that vanished in its flanking areas (vanished CSRs), a new compartment that emerged within the original compartment (emerged CSRs) and a compartment with a boundary that shifted into the flanking regions (shifted CSRs, Figs 2A and S6A). We applied k-means clustering on all CSRs and classified B-to-A CSRs into two vanished groups (229 CSRs), one shifted group (203 CSRs) and one emerged group (46 CSRs) (Fig. 2B). These four groups showed distinctive profiles of PC1 values and different types of compartments at their flanking regions (Fig. 2C and D). One vanished, one emerged and one shifted group were identified in A-to-B CSRs (Fig. S6B–D).

We next analysed other chromatin attributes in CSRs. Because chromatin accessibility is strongly correlated with active chromatin status [30], we expected an overall increase in ATAC signals in B-to-A CSRs. Surprisingly, the average ATAC signal in B-to-A CSRs remained stable throughout hepatic conversion, which was comparable with the level of ATAC signals in hepatocytes (Fig. 2E and F). When individual ATAC peaks were analysed, ATAC signals in B-to-A CSRs were significantly higher than those in unswitched B regions (Fig. 2G) and pre-existed even before 3TFs induction (Fig. 2H). Such pre-existing accessible sites were separated from franking regions by B-to-A CSR boundaries (Fig. 2E).

To further understand the pre-existing accessibility, we analysed individual accessible sites in B-to-A CSRs. K-means clustering of all ATAC peaks identified 2079 sites that pre-existed in B-to-A CSRs at Day 0 (Clusters 1 and 2) (Fig. 2I). Among these pre-existing accessible sites, 1137 were continuously open during hepatic conversion and remained accessible in hepatocytes (Cluster 1) and the other 942 showed reduced accessibility (Cluster 2). There were 1505 sites showing increased accessibility, with 420 newly gained (Cluster 3) and 1085 transiently increased (Cluster 4). The pre-existing and continuous accessible sites in Cluster 1 appeared to determine the overall high ATAC signals in B-to-A CSRs. Remarkably, Cluster 1 showed a large proportion of ATAC signals locating around promoters (356 of 1137), whereas ATAC peaks in Cluster 2 were mainly found at distal regions (Fig. 2J). Indeed, ATAC peaks in Cluster 1 overlapped with a larger number of promoters of induced hepatic genes (Fig. 2K). These data suggested that the pre-existing and continuous accessibility appeared to be strongly related with hepatic conversion. Interestingly, we found that 739, 634 and 856 pre-existing ATAC peaks were also accessible in ESCs, NPCs and cardiomyocytes, respectively. Unlike B-to-A CSRs, ATAC signals in A-to-B CSRs were diminished in association with the formation of inactive chromatin (Fig. S6E and F).

Given the unexpected finding of chromatin accessibility, we then analysed two major chromatin-remodeling complexes SWI/SNF and ISWI in regulating B-to-A CSRs by interfering with their core enzymes, Brg1 and Snf2h, respectively [31–33]. Brg1 was found to bind almost all accessible sites in fibroblasts (Fig. S7A) using published data [33]. Inactivation of Brg1 in fibroblasts led to decreased chromatin accessibility at these sites (Fig. S7B and C). Markedly, short hairpin RNA (shRNA) targeting Brg1 impaired iHep formation from fibroblasts (Fig. S7D and E). Because 3TFs transduction induced significant changes in chromatin accessibility at 48 hours, a Brg1 inhibitor PFI-3 [34] was applied within the first 48 hours to confine the inhibitory effect on chromatin-accessible sites. Remarkably, a dose-dependent inhibition of iHep formation was observed after PFI-3 treatment within the first 48 hours (Fig. S7F). By contrast, Snf2h showed a limited effect on chromatin accessibility [33] and hepatic conversion (Fig. S7G and H). These results supported a role for Brg1 in regulating chromatin accessibility during hepatic conversion.

We next analysed histone markers in B-to-A CSRs. H3K27ac was increased and H3K27me3 was reduced as expected along with the switch from compartment B to A (Fig. S8A–D). Notably, H3K27ac and H3K27me3 modifications appeared

to be demarcated by B-to-A CSR boundaries (Fig. S8A and C). We further annotated H3K27ac and H3K27me3 on the four clusters of chromatin-accessible sites. H3K27ac was increased at pre-existing and continuous accessible sites in Cluster 1 that were enriched with induced genes (Fig. S8E). H3K27me3 that covered broader regions over ATAC peaks was reduced in all clusters (Fig. S8E). The dynamic regulation of histone modification suggested that B-to-A CSRs may belong to facultative heterochromatin [35]. H3K4me1 and H3K4me3, markers related to facultative heterochromatin, were additionally analysed. H3K4me1 showed comparable kinetics to H3K27ac, whereas H3K4me3, frequently located at promoter regions, was observed mainly at ATAC peaks in Cluster 1 (Fig. S8E). In A-to-B clusters, H3K27ac levels were reduced (Fig. S8F and G), whereas H3K27me3 levels remained low during hepatic conversion (Fig. S8H and I).

These data together showed that B-to-A CSRs possessed several interesting chromatin attributes in fibroblasts, including pre-existing and continuous chromatin accessibility at hepatic genes, and inactive histone markers with low H3K27ac and high H3K27me3, illustrating a mosaic chromatin status. Moreover, these chromatin attributes were apparently segregated from flanking regions by B-to-A CSR boundaries. As examples, such unique chromatin organization and mosaic status in B-to-A CSRs was illustrated in hepatic gene *Vnn1* (Fig. 2L).

### Related positioning of B-to-A CSRs and loops in chromatin

**Figure 3.**
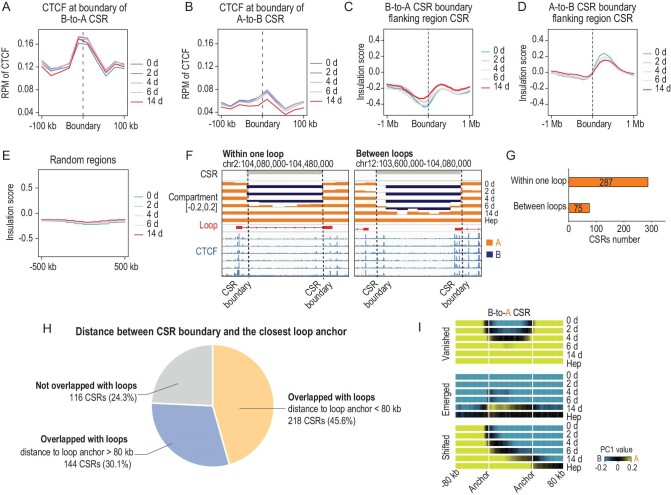
CTCF binding was adjacent to loop anchors. (A and B) Distribution of CTCF around the boundary (±100 kb) of B-to-A CSRs (A) and A-to-B CSRs during hepatic conversion (B). (C–E) Distribution of insulation scores around the boundary (±1 Mb) of B-to-A CSRs (C) and A-to-B CSRs (D), and random regions (E) during hepatic conversion. The dashed lines indicate the CSR boundary. We plotted the insulation score around the CSR boundary. The data were organized to show the CSR regions on the right of the panel and the flanking regions on the left of the panel. (F) Representative examples of CSRs located within a loop, or between two loops. Compartmentalization, loop and CTCF binding are shown. (G) Number of CSRs located within a loop or between two loops. (H) Pie graph showing the proportion of CSRs according to the distance between the CSR boundary and its adjacent loop anchor. (I) Average heat map showing compartmentalization around loop anchors. Loop anchors adjacent to B-to-A CSR boundaries (within 80 kb) were selected. PC1 values were profiled at regions between a pair of loop anchors and flanking regions.

The finding that chromatin accessibility and histone modifications in B-to-A CSRs were separated from flanking regions suggested a likely insulation at the boundaries. CTCF was reported as a key chromatin-structure-associated protein regulating insulators and barrier elements in the genome [36–38]. CTCF-binding sites were found conserved during hepatic conversion (Fig. S9A and B). Notably, strong CTCF occupancy was identified around B-to-A CSR boundaries (Fig. 3A and B), whereas CTCF binding was relatively weak around boundaries of A-to-B CSRs (Fig. 3B). In total, 855 of 956 (89.4%) B-to-A CSR boundaries showed CTCF binding, which was higher than its binding to random regions (31.7%) (*P *< 2.2e-16, Fisher's exact test) and compartment boundaries (72.3%) (*P *< 2.2e^-16^, Fisher's exact test). The insulation by CSR boundaries was further validated using the insulation score [39] (Fig. 3C–E). These data suggested a role for CTCF in association with the insulation of B-to-A CSR boundaries.

It was shown that the insulation of CTCF was achieved by forming chromatin loops [20,40]. However, chromatin compartmentalization was reported to be largely independent of the binding of CTCF [41,42]. The existence of CTCF at the boundary of B-to-A CSRs raised the question of whether there was an unidentified relation between chromatin organization between compartments and loops [41,43–45]. When spatial distributions of B-to-A CSRs and loops were aligned, we found that

287 (60.0%) B-to-A CSRs were located within a loop and 75 (15.7%) between two loops (Fig. 3F and G). Notably, 218 (45.6%) B-to-A CSR boundaries were situated close to loop anchors (Fig. 3H) within 80 kb. These results suggested that B-to-A CSRs were adjacent, but not identical, to loops in spatial distribution. We also analysed the relationship between CSRs and loop domains [18]. Overall, 749 loop domains were identified and most B-to-A CSRs (463 of 478, overlapped regions/CSR > 0.8) did not represent loop domains.

To further reveal whether loop anchors were involved in insulating B-to-A CSRs, we visualized compartment switches within loop anchors. The kinetics of PC1 values was profiled between loop anchors that were adjacent to B-to-A CSR boundaries. We expected that PC1 values would be changed within loop anchors if the switch of B-to-A CSRs was insulated by loop anchors. Indeed, compartment regions with changed PC1 values were restrained within loop anchors (Fig. 3I). Together, these data suggested that loop anchors were closely located to B-to-A CSR boundaries in insulating compartment switches.

### Foxa3 binds chromatin-accessible sites in B-to-A CSRs

We next analysed how chromatin organization and mosaic status interacted with 3TFs during hepatic conversion. At Day 2, Foxa3-binding sites appeared to overlap largely with Gata4 and Hnf1a (Fig. S10A and B). In addition, because Foxa3 was previously reported as the most important factor for hepatic conversion [46,47], we focused on Foxa3 and compartment switches.

**Figure 4.**
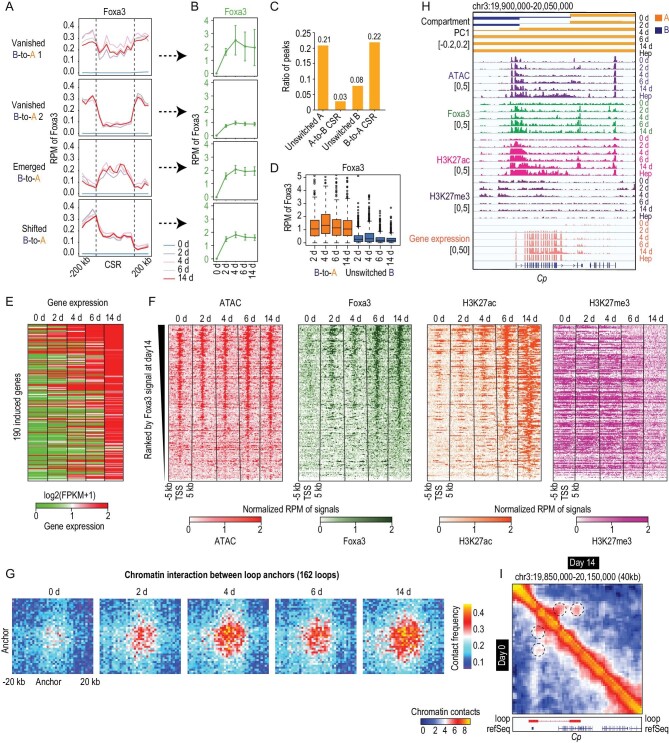
Foxa3 binds chromatin-accessible sites in B-to-A CSRs. (A) Foxa3 binding at four groups of B-to-A CSRs and their flanking regions. (B) Broken line graph showing the kinetics of Foxa3 binding in four cluster B-to-A CSRs. Data are presented as mean ± s.d. (C) Histogram showing the proportion of remaining Foxa3 peaks relative to all Foxa3 peaks in unswitched compartment A, A-to-B CSRs, unswitched compartment B and B-to-A CSRs, respectively. (D) Box plots of Foxa3 binding in B-to-A CSRs and unswitched compartment B regions. (E and F) Heat maps of gene expression (E), Foxa3 binding, ATAC signals, H3K27ac and H3K27me3 modification (F) around induced genes (transcription start sites ±5 kb) in B-to-A CSRs. (G) Chromatin interaction between loop anchors. Loops with induced genes at the anchor regions were selected and regions of anchors ±20 kb are shown. (H) Representative examples of chromatin status, compartmentalization, ATAC signal, Foxa3 binding, H3K27ac, H3K27me and gene expression in a B-to-A CSR where the hepatic gene *Cp* is located. (I) Representative example of chromatin interactions around hepatic gene *Cp.* Heat map showing the chromatin interaction at 40-kb resolution.

The binding of Foxa3 to B-to-A CSRs was dramatically increased at Day 2 and remained at high levels from Days 4–14 (Fig. 4A and B). B-to-A CSRs showed more sites constantly bound by Foxa3 than unswitched compartment B (Fig. 4C). Foxa3 binding (Fig. 4B) was concomitant with or earlier than compartment switches in B-to-A CSRs (Fig. 2D). When compared with the kinetics of histone modifications, Foxa3 binding (Fig. 4B) appeared to be 2–4 days earlier than the increase in H3K27ac and the reduction in H3K27me3 in B-to-A CSRs (Fig. S8B and D), supporting a role for Foxa3 in triggering changes in chromatin organization [29,48]. By contrast, Foxa3 binding to A-to-B CSRs was increased at Day 2 and then diminished at Day 6 (Fig. S10C and D).

The pre-existing and continuous accessible sites in B-to-A CSRs raised the question of whether Foxa3 binding was correlated with pre-existing accessibility. By analysing the binding sites of Foxa3, we found that Foxa3 strongly bound to pre-existing and continuous chromatin-accessible sites in all B-to-A CSRs at Day 2 and most of the Foxa3 binding remained stable during hepatic conversion (Figs 4C and S11A–C). By contrast, the binding of Foxa3 was much lower in unswitched compartment B regions (Fig. 4D). Specifically, Foxa3 binding was increased at induced genes and was associated with pre-existing chromatin accessibility at these genes (Fig. 4E and F). Following Foxa3 binding, pre-existing accessible sites gained H3K27ac and eliminated H3K27me3 (Fig. 4F). In line with previous findings that gene activation was associated with increased chromatin interaction [49], 100 of 190 induced genes in B-to-A CSRs were found located at loop anchors, and increased chromatin interactions were identified in these loops (Fig. 4G). Together, these findings suggested that pre-existing accessibility in B-to-A CSRs was consistent with increased Foxa3 binding.

To summarize the kinetics of chromatin change, a B-to-A CSR that contained the hepatocyte-expressed gene *Cp* was presented as an example (Fig. 4H and I). This CSR was located in compartment B and showed pre-existing and continuous accessibility in fibroblasts. Foxa3 bound to chromatin-accessible sites and remained at the high level until Day 14. Following with Foxa3 binding, this region was gradually switched from compartment B to A, accompanied by increased H3K27ac, reduced H3K27me3 and enhanced intra-loop interaction. Notably, the expression of *Cp* was induced during this process. Comparable kinetics was also exemplified by another B-to-A CSR where *Arhgef28* resided (Fig. S11D and E).

### Mosaic status of switchable compartment B in other cell-identity conversions

Our results demonstrated that B-to-A CSRs, insulated by CTCF occupancy, possessed a mosaic chromatin status in fibroblasts, i.e. located in a repressive compartment with low H3K27ac and high H3K27me3 and, meanwhile, retained pre-existing accessibility to factors of hepatic lineage. We asked whether such a mosaic status was specific to hepatic conversion or was also involved in conversion to other cell identities.

**Figure 5.**
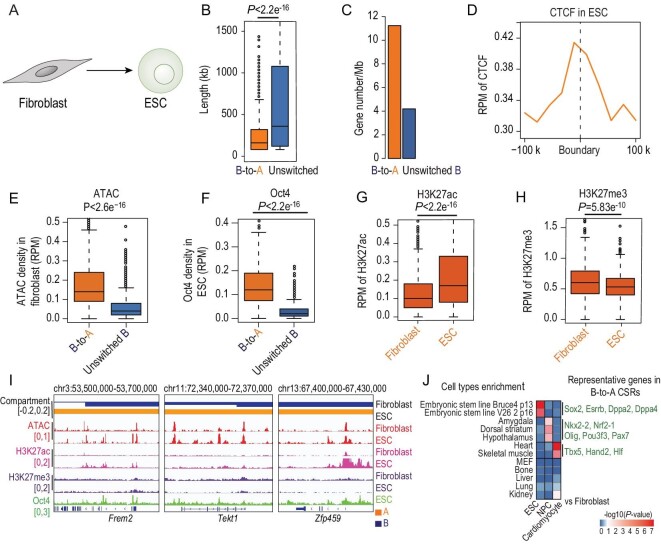
Mosaic status in switchable compartment B from fibroblasts to pluripotent stem cells. (A) Fibroblasts and ESCs are compared to analyse the mosaic status in switchable compartment B. (B) Genomic length of B-to-A CSRs and unswitched compartment regions. (C) Gene density of B-to-A CSRs and unswitched B regions. (D) Distribution of CTCF binding at boundaries of B-to-A CSRs in pluripotency conversion. Boundary ±200 kb is shown. (E and F) Box plots of average ATAC signal (E) and Oct4 binding level (F) in B-to-A CSRs and unswitched compartment B regions. Wilcoxon rank sum test was applied. (G and H) Box plots showing average H3K27ac (G) and H3K27me3 (H) in B-to-A CSRs in pluripotency conversion. (I) Representative examples showing compartmentalization, chromatin accessibility, H3K27ac, H3K27me3 and Oct4 distribution around pluripotency genes, *Frem2*, *Tekt1* and *Zfp459*. (J) B-to-A CSRs were determined by comparisons of fibroblasts with ESCs, NPCs and cardiomyocytes, respectively. Induced genes that are in these B-to-A CSRs were analysed for the enrichment of cell-type-specific genes by online software Enrichr. Heat map showing *P*-values of the enrichment for different cell-type-specific genes. Key genes for ESCs, NPCs and cardiomyocytes are shown next to the heat map in green. *P*-values were calculated using Fisher's exact test.

To that end, we first characterized the reprogramming from fibroblasts to pluripotent stem cells and focused on analysing chromatin accessibility, CTCF binding, cell-type-specific transcription factors and gene expression in switchable compartment B. Because of the similarity of induced pluripotent stem cells and embryonic stem cells (ESCs) [26], published data of ESCs [50] were analysed (Fig. 5A). The median length of B-to-A CSRs related to pluripotency induction was 160 kb with a gene density of 11 genes/Mb (Fig. 5B and C), which was comparable to B-to-A CSRs of iHep. CTCF occupancy was identified around the boundaries of these B-to-A CSRs (Fig. 5D). Markedly, B-to-A CSRs related to pluripotency induction showed 3752 pre-existing accessible sites in fibroblasts (Fig. 5E). Remarkable binding of Oct4, the key factor for pluripotency induction [51], was identified in these B-to-A CSRs as well (Fig. 5F). Increased H3K27ac and decreased H3K27me3 were found during pluripotency induction (Fig. 5G and H), which was in line with the induced expression of pluripotency genes locating in these B-to-A CSRs (Fig. 5I and J), such as *Frem2*, *Tekt1* and *Zfp459* [52].

In addition to pluripotency induction, we analysed conversions to neural cells and to cardiomyocytes from fibroblasts. Published data of NPCs [50] and cardiomyocytes [53], which were highly comparable to iN and iC [54,55], were applied (Fig. S12). Notably, B-to-A CSRs related to either neural conversion or cardiomyocyte conversion were occupied by CTCF at boundaries (Fig. S12D and L). High chromatin accessibility (3811 and 3650 pre-existing ATAC sites for neural conversion and cardiomyocyte conversion, respectively) was detected inside these B-to-A CSRs

(Fig. S12E and M). Moreover, these B-to-A CSRs showed increased binding of key lineage transcription factors, Ascl1 of neural induction and Tbx5 of cardiomyocyte induction (Fig. S12F and N), respectively. Accompanied by augmented H3K27ac (Fig. S12G and O) and reduced H3K27me3 (Fig. S12H and P), cell-type-specific genes were induced (Fig. 5J). These data supported that the mosaic status in switchable compartment B regions is associated with multiple lineage conversions from fibroblasts.

Finally, to extend the finding of mosaic status in cells other than fibroblasts, we analysed the conversion from human B-cells to macrophages using published data [56]. CTCF occupancy was also found at the boundaries of B-to-A CSRs in this lineage-conversion system (Fig. S13A and B). Accordingly, 1969 chromatin-accessible sites were found in B-to-A CSRs of B-cells during the conversion to macrophages (Fig. S13C). Moreover, increased CEBPA binding was identified in these B-to-A CSRs, accompanied by an increase in H3K27ac and gene expression after conversion to macrophages (Fig. S13D–F). These results together suggested that the mosaic status exists in cell types other than fibroblasts.

## DISCUSSION

In this study, we characterized cell plasticity by dissecting high-order chromatin organization during transdifferentiation from fibroblasts to hepatocytes. The CSR was identified as a chromatin structure of local compartment switch, demonstrating unique length and gene density. Specifically, B-to-A CSRs possessed a mosaic chromatin status showing pre-existing accessibility in the repressive compartment in fibroblasts. Such mosaic status was demarcated by CTCF occupancy and enhanced the binding of Foxa3, in association with chromatin activation and cell-type-specific gene expression. Our findings thus suggested mosaic chromatin status as a possible framework to understand the intrinsic attribute of cell plasticity.

Our data describe a genomic region that featured as transcriptionally inactive but was permissive to regulatory factors during hepatic conversion. In addition to pre-existing accessibility, active histone modifications have been reported to pre-paint the repressive chromatin and prepare a permissive state [15,57]. A permissive state was often observed during embryonic development [58], e.g. the specification from definitive endoderm to liver and pancreas [59]. Besides hepatic genes, we found pre-existing accessible sites in compartment B regions related to multiple lineage genes. The pre-existing accessibility at genes of multiple cell lineages thus appeared to be a general epigenetic basis underlying cell plasticity. It has been previously reported that ∼37% of DNA hyper-sensitive sites in differentiated cells were found in ESCs [58]. The pre-existence of chromatin-accessible sites in compartment B might also be partially explained as developmental remnants. It is reasonable to speculate that such retained accessibility in lineage genes might be a fundamental epigenetic attribute to facilitate reprogramming. However, it was unclear by which mechanisms these lineage genes were maintained as accessible. It is plausible that the SWI/SNF complex may be involved, as these factors were previously reported as being essential in activating chromatin accessibility [30,60,61]. Moreover, the dynamic regulation of repressive histone modification suggested that B-to-A CSRs belong to facultative heterochromatin. It is interesting to speculate that protein complexes decompacting heterochromatin might be also involved.

In our study, pre-existing chromatin accessibility may endow a permissive state to repressive compartment B regions, increasing the accessibility to pioneer factors, such as Foxa3, to induce compartment switch and hepatic genes activation. Indeed, pioneer factor Foxa3 was found to bind strongly and stably to all B-to-A CSRs, either with or without induced genes, during the hepatic conversion. Transcription factors are important in reorganizing chromatin structures [27,62]. During the conversion from B-cells to iPSCs, Stadhouders *et al.* reported that transcription factors bound at the TAD boundary to modify the insulation, therefore facilitating the formation of iPSCs [27]. Moreover, transcription factors MyoD mediated chromatin interactions between promoters and enhancers during the induction of myoblasts from fibroblasts [62], which led to the repression of fibrotic genes and the induction of myogenic genes. Intriguingly, we also found that the binding of Foxa3 appeared to be largely facilitated by pre-existing accessibility in B-to-A CSRs, implying an underappreciated feature of *cis*-elements in active interaction with pioneer factors. Besides B-to-A CSRs, Foxa3 bound to hepatic genes located in other genomic regions. As we previously reported, chromatin opening at these sites may lead to occupancy of the SWI/SNF complex, which on the other hand activates the ATM-p53-dependent chromatin-remodeling checkpoint to safeguard fibroblast identity [29].

Compartments, TADs and loops were thought to be hierarchical structures of chromatin [23,63]. TADs are largely cell-type invariant, being physically and functionally isolated units of a genome [17,64]. Within TADs, there exist more defined structures, including sub-TADs [65], loops [18] or insulated neighborhoods [49], which are partially cell-type-specific and related to transcriptional regulatory events [5]. Recent studies revealed independent mechanisms for the establishment and maintenance of compartments and loops [41,42,44,66]. Loss of CTCF or cohesin complex binding led to the destruction of TADs and loops; however, compartments remained intact or even formed finer structures. These findings suggested that compartmentalization and loop extrusion likely depended on distinct mechanisms [41,42,44,45,67]. Intriguingly, our data suggested that compartment switch might happen at the level of the loop. Notably, loop anchors were located adjacent to B-to-A CSRs and seemed to insulate the compartment switch by constraining the range of CSRs, suggesting a possible interplay between compartments and loops. One prominent difference between our study and others was that we analysed the kinetics of cell-lineage conversion, while others focused on the loss of CTCF or cohesin in a defined cell type. It is possible that the loss of a loop anchor may not necessarily require alteration of the compartmentalization, which in a stable cell identity is likely maintained by other mechanisms [41,45,66]. However, in lineage conversion, the binding of transcription factors to pre-existing accessible sites that were ambushed in the repressive compartment may establish an active chromatin status and facilitate loop extrusion and compartment switch. Given that CTCF deletion impaired cell proliferation [68], to reveal the exact molecular regulations would require experimental design other than interfering with CTCF. It is nevertheless intriguing to speculate that such dynamic changes in compartmentalization were coupled with loops. Whereas an active loop restrains interactions of regulatory chromatin elements only within loops, long-range interactions between active loops may contribute to compartmentalization. A prediction derived from our study therefore would be that the formation of cell-type-associated compartments might be based on the unit of loops. As partial support for this prediction, the majority of compartment boundaries are located close to loop anchors in a given type of cells [69].

Together, we provided a systematic analysis of chromatin changes during hepatic conversion. We proposed that pre-existing chromatin accessibility at compartment B regions delineated an intrinsic attribute for cell plasticity, which could be applied to reprogramming of multiple cell identities. Interestingly, pre-existing chromatin accessibility as an intrinsic attribute for gene activation has been associated with various biological processes, such as T-cell differentiation, response to glucocorticoid hormones and the dedifferentiation of hepatocytes after injuries [13–15,70]. In development and regeneration, pre-existing accessibility is necessary to properly unfold these processes. However, it is unlikely that pre-existing accessibility specifically prepares cells for reported reprogramming systems *in vitro*. It would be interesting to further determine the functional characteristics of such a mosaic chromatin status *in vivo*, which might lead to unrecognized functions in development and injury responses.

## MATERIALS AND METHODS

Materials and methods, including statements of data availability and any associated accession codes and references, are available in Supplementary Information.

## Supplementary Material

nwab230_Supplemental_Files
