# Supplementary material for: Pre-existing chromatin accessibility of switchable repressive compartment delineates cell plasticity
Source: Natl Sci Rev. 2021 Dec 31;9(6):nwab230. doi: 10.1093/nsr/nwab230 (PMC9249582; doi:10.1093/nsr/nwab230)
Supplement: nwab230_Supplemental_Files [file nwab230_supplemental_files.zip › Manuscript_Supplementary_Information_revise_20211129.docx]

**Supplementary Information of**

“Pre-existing chromatin accessibility of switchable repressive compartment delineates cell plasticity”

Xiaolong Ma^1^†, Xuan Cao^2^†, Linying Zhu^1^†, Ying Li^2^, Xuelong Wang^2^, Baihua Wu^1^, Gang Wei^2^* and Lijian Hui^1,3,4,5,6^*

1, State Key Laboratory of Cell Biology, Shanghai Institute of Biochemistry and Cell Biology, Center for Excellence in Molecular Cell Science, Chinese Academy of Sciences; University of Chinese Academy of Sciences, Shanghai 200031, China

2, CAS Key Laboratory of Computational Biology, Shanghai Institute of Nutrition and Health, Chinese Academy of Sciences, University of Chinese Academy of Sciences, Shanghai 200031, China

3, Institute for Stem Cell and Regeneration, Chinese Academy of Sciences, Beijing 100101, China

4, Bio-Research Innovation Center, Shanghai Institute of Biochemistry and Cell Biology, Suzhou 215121, Jiangsu Province, China

5, School of Life Science and Technology, ShanghaiTech University, 100 Haike Road, Shanghai 201210, China

6, School of Life Science, Hangzhou Institute for Advanced Study, University of Chinese Academy of Sciences, Hangzhou 310024, China

†, These authors contributed equally to this work

*Correspondence to: Lijian Hui (ljhui@sibcb.ac.cn), Gang Wei (weigang@picb.ac.cn)

**This file contains:**

Materials and Methods

Tables S1 to S4

Legends of Fig. S1 to S13

Reference

Materials and Methods

**Cell culture and iHep cell induction**

Mouse tail fibroblast (TTF) cell line was established before from p19^Arf-/-^ mice [1]. TTFs were cultured and maintained in DMEM 10x basic medium (Gibco) with 15% FBS (Gibco). Reprogramming of TTF into induced functional hepatocyte was performed in a similar way to previous methods with improvement. First, the multiplicity of infection (MOI) of each virus was increased to 3. Then, for the induction medium, we elevated the concentration of TGFα to 40ng/mL, EGF to 40ng/mL and Dexamethasone (Dex) to 10μM. Brieﬂy, 5x10^5^ TTFs were seeded in a 10cm dish and overexpressed Foxa3, Gata4 and Hnf1a by lentivirus. Harvesting was done at the indicated time points, day 0, 2, 4, 6 and 14, by cell scraper. All cell lines were routinely tested for mycoplasma contamination.

To detect the impact of inhibiting Brg1 to hepatic conversion, we added the PFI-3 (Selleck) at the concentration of 2μm, 5μm and 10μm into the supernatant for the first 2 days of hepatic conversion.

**Molecular cloning and lentivirus production.**

The constructions of 3TFs were described previously[1]. We also replaced GFP at pWPI.1 plasmid (Addgene) into mCherry, ZsGreen and HSA respectively by ClonExpress® MultiS One Step Cloning Kit from Vazyme. Primer used were listed in Table S2.

293FT cells are used for lentivirus production. 15μg constructed pWPI plasmids, 10μg psPAX2 (Addgene) and 5μg pMD2.G (Addgene) plasmids are co-transfected into 293FT. After 48 hours incubation, supernatants with lentivirus were collected by the 0.45μm filter and stored at -80°C.

**Immunofluorescence**

To perform immunofluorescence staining, cells were washed by PBS once and fixed by 4% paraformaldehyde at room temperature for 15min. Cells then were incubated with 0.25% Triton X-100 (Sigma) for 15 min, washed by PBS for three times and blocked by 3% BSA (Solarbio) for 1 hour. Following that, cells were incubated with primary antibodies at 4°C overnight, washed by PBST (Tween-20, 1:1000) for three times and incubated with appropriate second antibody in the dark. Cell nucleus were stained by DAPI at room temperature for 5 min and washed by PBST for three times. The primary and second antibodies were suspended in PBS with 3% BSA. Images were captured by confocal microscope (Zeiss LSM880 Ariyscan). Antibodies used in immunofluorescence were listed in Table S3.

**RNA-seq**

RNA was extracted from iHep cells at different time points and liver with a Trizol (Invitrogen) according to the manufacturer’s instructions and quantified with a NanoDrop 2000 spectrophotometer (ThermoFisher) respectively. 1μg RNA was used for reverse transcription. Libraries were prepared with an Illumina TruSeq Stranded mRNA Library Preparation Kit, and single-end sequencing (100bp) was performed on an Illumina HiSeq2000 instrument (PICB).

**ATAC-seq**

ATAC-seq assays were performed as described before [2], and two biological replicates were performed for each time point. Cells were collected by trypsin attenuated by PBS (trypsin, 1:5). Nuclei isolated from 50,000 counted cells were used for transposition reaction with Tn5 transposase (Vazyme Biotech). 10 PCR cycles were performed for library setup for each reaction mix using high-fidelity 2x PCR Master Mix (New England Biolabs) using primers with unique barcodes (Vazyme Biotech). Pair-end sequencing (150bp x 2) was performed on Illumina HiSeq X Ten instrument (CloudHealth Genomics Ltd.).

**Primary hepatocyte isolation**

Primary hepatocytes were isolated from p19^Arf-/-^ mice by standard two-step collagenase perfusion. Then, cells were filtered by 70μm filter (BD Bioscience) and purified by Percoll (Sigma) buffer with a series of low-speed centrifugation as previously described [3]. After removing the supernatant, resuspend the cells with 1% formaldehyde for cross linking at room temperature, which was used for ChIP experiment.

**ChIP-seq**

For ChIP-seq assay of transcription factor, chromatin binding protein and histone modifications, cells at different time points were collected by cell scraper for experiment. 2x10^6^ cells are used for ChIP of H3K27ac and H3K27me3, and 10^7^ cells are used for ChIP of Foxa3, Gata4, Hnf1a and CTCF. Antibodies used in ChIP experiment are listed in the supplementary table. Cells are cross-linked by 1% formaldehyde for 10 min at room temperature, followed by glycine quenching for 5 min, and collected by cell scraper. Nuclear extracts were collected and washed in PBS. Sonicate the chromatin in nuclear extracts to fragments by ultrasonication (Covaris S220). Primary antibodies were used for immunoprecipitation in sonication supernatant by incubation at 4°C overnight. Protein G Dynabeads (Life Technologies, 10004D) were used for pulling down antibody-chromatin complex followed by washing and eluting. Beads with antibody and target chromatin were reversed for cross-linking, and samples were treated with proteinase K. MinElute PCR purification kit (Qiagen) was used for ChIP fragments collection. Qubit (Life Technologies) was used for ChIP DNA quantification. NEBNext Ultra DNA Library Prep Kit was used for ChIP-seq library preparation. Single-end sequencing (100bp) was performed on the Illumina HiSeq2000 instrument (PICB). Antibodies used in ChIP-seq were used in Table S3.

**In Situ Hi-C**

Five million cells were cross-linked in 1% formaldehyde for 10 minutes at room temperature to immobilize the nucleus. After nuclei being permeabilized, DNA was cut with restriction enzyme DpnII (NEB), and the end of the restriction fragment was labeled with biotin-14-dATP (Life Technologies). Then, the biotinylated products were used for proximity ligation. After the reversed cross-linking of the ligated biotinylated DNA, it was purified and broken into about 400bp fragments by ultrasonic treatment (Covaris S220). The ligation products with biotin-labeled ligaments were enriched by using MyOne streptavidin anti-biotin protein C1 Dynabeads (Life Technologies) and followed by Illumina double-ended sequenced ligation. The sequencing libraries were generated using 11-12 PCR cycles. Paired end sequencing (150bp x 2) was performed on Illumina HiSeq X Ten instrument (Annoroad Ltd.).

**RNA-seq data processing**

Reads of RNA-seq samples were mapped to the mouse reference genome (mm10) using TopHat v.2.1.1 [4]. The gene expression level for each sample was calculated using Cufflinks v.2.2.1 [5] as FPKM based on Refgene annotation from the UCSC genome browser. We used htseq-count [6] to count reads on genes. Differential expression analysis was performed using R package, DESeq [7]. Genes were considered differentially expressed, which were induced or silenced from day 0 to day 14, if DESeq p-value <0.05, FPKM>1 in day 0 or day 14 sample and | log2(fold changes) | >= 0.58. Gene ontology analysis on top 3000 differentially expressed genes of day 0 and day 14 were performed by Metascape [8].

**ChIP-seq data and ATAC-seq data processing**

CutAdapt v.1.16 [9] was used to remove adaptor sequences from raw sequencing single-end 100-bp reads. Reads were mapped to the mouse reference genome (mm10) using bowtie v.1.1.1 [10]. Duplicate reads were removed using in house script. Peaks were called using macs v.1.3.7.1 [11] with -10*log10 (p value) > 200 were kept. Foxa3 ChIP-seq and ATAC-seq samples were quantile normalized to remove the bias across the samples by using haystack_bio v.0.5.5 (https://github.com/pinellolab/haystack_bio) [12]. BedGraph files were normalized for total mapped read counts and signal calculated as per million per kilo-base(rpm). Profiles of ChIP-seq and ATAC-seq signals at individual regions of interest were created by quantifying the signal in each bin from BedGraph files. We removed repetitive regions according to the list provided by ENCODE (https://www.encodeproject.org/search/?type=Annotation&annotation_type=blacklist) before calling peaks and quantifying chromatin accessibility. Meta-profile plots were used to display the average ChIP-seq and ATAC-seq signal of interested regions by calculating the mean ChIP-seq and ATAC-seq signal profile across the interested regions. Clustering individual peaks in B-to-A CSRs was performed by cluster 3.0 based on average chromatin accessibility of each peak, and 4 groups were identified and shown in Figure 2I. Our results showed that pre-existing and permissive ATAC peaks functionally related to gene promoters and induced genes, which suggested that these peaks were not a random event during hepatic conversion. Integrative Genomics Viewer (IGV) was used for data visualization in genome [13,14]. In Figure 5E, Foxa3 signal at day 0 was not shown because of the low signal which made the sample inappropriate to perform quantile normalization and compare to other time points. We also analyzed our ATAC-seq data by applying the pipeline by Reske et al. [15], which included process of normalizing library complexity, and found that both of methods produced comparable conclusions.

**Hi-C data processing pipeline**

**Reads mapping**: Hi-C sequencing data were 150bp paired-end reads. Paired-end reads were fully mapped to the mouse reference genome (mm10) using bowtie v.1.1.1 [10] with parameters: -m 1 -k 1. Hi-C reads were chimeras that were composed of DNA from two or more loci. If the ligation junction happened to fall within the sequenced fragment, these reads could not fully be mapped to the reference genome. We filtered out any reads that were not fully mapped to the genome and truncated the reads by the DpnII restriction site. Truncated reads that were longer than 20bp were mapped back to the genome. After alignment, reads in above two mapped files were paired by the name of reads.

**Validation and filtering of read pairs**: Hi-C read pairs for which both ends were successfully aligned to the genome were further segregated into two categories. For some read pairs, each read in a read pair was aligned to a single site in the genome. Other read pairs were “chimeric”: one read arose from ligation products between locus A and locus B, and the other reads were mapped to either locus A or locus B[16]. These chimeric read pairs were kept for further analysis and all other chimeric read pairs were filtered out. At the ligation step, the two digested ends cut by one enzyme restriction site had more probability to be ligated together. Using 4-cutter restriction enzyme DpnII, size selecting for 300–500 bp used in Hi-C experiment, we filtered out the proximal ligation with the distance between 2 reads < 500bp in a read pair. The duplication removal step was to discard the read pairs which were aligned to the same genome position.

**Construct and normalize Hi-C contact matrix**

We divided the linear genome into 40kb bins and counted the contacts between each bin pair of all samples. Each entry in the matrix containing a number of contacts between locus i and locus j was denoted as $\mathbf{Mij}$. There have been various approaches in normalizing the contact matrix in order to eliminate bias. The original method to address matrix normalization was “vanilla coverage normalization (VC normalization)” [17]. For each entry$\mathbf{Mij}$, VC normalization had the row and column specific factor termed **Ri** and $\mathbf{Cj}$ which was calculated by summing the counts in a row or a column and taking the reciprocal separately. Therefore, the normalized entry $\mathbf{M*ij}$ was $\mathbf{Ri*Mij*Cj}$.

**Identify A and B Compartments**

**Observed/expected (O/E) matrix**: We calculated O/E matrix as in Lieberman-Aiden et al.[17]. At 40kb resolution, we calculated the total number of contacts at a given genomic distance **d** between locus pairs termed as **I(d)**. Each chromosome contributed **Lc-d** to the number of contacts at distance **d**, where **Lc** was the length of the chromosome. The expected number of contacts for each entry **Mij** was therefore the result of dividing the total number of contacts at distance **d=|i-j|** by the number of contacts observed of **Mij**.

**Pearson’s correlation matrix**: The Pearson’s correlation matrix was computed by Pearson correlation coefficient on O/E matrix.

**Identification of compartments using principal component analysis (PCA)**: To identify compartments, we performed principal component analysis (PCA) of correlation matrix of intra-chromosomes [17]. We used the first principal component (PC1) to differentiate A/B compartments and confirmed the active A and repressive B compartment by the reference active histone ChIP-seq (such as H3K27ac ChIP-seq) or ATAC-seq data.

**Visualization of the difference of Hi-C interaction frequencies between two samples**: To generate and visualize interaction frequency heatmaps of whole chromosomes, Hi-C matrices at 40kb resolution were imported to the software Juicebox (version 1.11.08). For visualization of the difference of Hi-C interaction frequencies between 0d and 14d sample, we used Juicebox to generate the ratio of 14d/0d interaction frequency matrices. As shown in Fig. 1E, Fig. S3F and G, we used a similar method described above.

**Call Compartment Switchable Regions (CSRs)**

After identifying the A/B compartments at the resolution of 40 kb, we called continuous compartment switchable regions (CSRs) among all samples.

The first step was to obtain the bins that had different state of PC1 values (A/B or B/A) in two samples. The second step was to classify these bins into B-to-A and A-to-B bins. For B-to-A bins, we connected the continuous bins allowing one bin gap and defined these regions as B-to-A CSRs. A-to-B CSRs were called in the similar way. During the process of trans-differentiation, we called CSRs between samples of any two time points, and defined the final CSRs regions by merging all these regions.

To compare the difference of compartmentalization between fibroblast, iHep cells and hepatocytes, we follow the method in calling CSRs, and only analyzed these three samples.

**Classify CSRs**

In order to classify CSRs by both flanking regions, we divided the genome into CSRs, unswitched compartment A and unswitched compartment B regions. For each CSR, we searched their flanking regions and classified them into 3 groups. Group I was CSRs with both flanking regions which were unswitched compartment A (A_CSR_A), and we termed them as vanished B-to-A CSRs or emerged A-to-B CSRs. Group II was termed as emerged B-to-A CSRs or vanished A-to-B CSR, both flanking regions of which were unswitched compartment B (B_CSR_B). In group III, one flanking region was unswitched compartment A and another was unswitched compartment B (A_CSR_B and B_CSR_A), and these CSRs were termed as shifted B-to-A CSRs or shifted A-to-B CSRs. In order to get the same direction of PC1 change along shifted CSR and their flanking regions (A_CSR_B and B_CSR_A), we reversed the direction of B_CSR_A shifted CSRs to A_CSR_B.

For clustering the CSRs, we divided each CSR into 10 equal bins, mapped average PC1 values to bins and then performed k-means clustering.

**Call TAD**

TADs were called by Topdom software as previously described [18]. We called TAD based on normalized contact matrix at 40kb resolution and set the window size as 10. We captured 3420, 3445, 3490, 3323 and 3432 TADs at day 0, 2, 4, 6 and 14, respectively. TAD and associated contact matrix were visualized in WashU EpiGenome Browser [19].

**Call Insulation Score**

Insulation score was called by cworld software (<https://github.com/dekkerlab/cworld-dekker>) as previously described [20] at 40kb resolution. The insulation score was calculated by sliding a 500kb squared matrix along the diagonal of the interaction matrix and a 200kb window was used for calculating the delta vector for each chromosome.

**Call loop**

For the loop calling we used the similar approach as in Peter Hugo al [21]. For each intra-chromosome, we mapped Hi-C reads to fragment space matrix and normalized matrix by VC normalization. To make fragment space matrix, we divided linear genome to fragments based on the DpnII restriction sites and used overlapping windows with a size of 50 fragments and a step size of 5 fragments corresponding to a median genomic size of 20kb.

Loops in Hi-C contact matrix showed enrichment in reads count over the background. The background of contact matrix followed a monotonically decreasing pattern along the vertical diagonal which was a function of the distance to the site of interest. To build the monotone decreasing background model, we used isotonic regression algorithm and PAVA (Pool Adjacent Violators Algorithm) to solve this problem. Isotonic regression model:$\mathbf{f(x)=}\sum_{\mathbf{i=1}}^{\mathbf{n}} \mathbf{w}_{\mathbf{i}}\mathbf{*}\left( \mathbf{y}_{\mathbf{i}}\mathbf{-}\mathbf{x}_{\mathbf{i}} \right)^{\mathbf{2}}$, where $\mathbf{w}_{\mathbf{i}}$was the weight and$\mathbf{y}_{\mathbf{i}}\mathbf{<}\mathbf{y}_{\mathbf{i+1}}$. For each entry in fragment space matrix, we counted the observed reads number and calculated O/B ratio which was the ratio of observed reads number over background. Firstly, we picked out entries that the threshold of O/B ratio was higher than 1.5 and the minimal reads number was 60 as candidate loops. Due to the way we used to make the fragment space matrix and one loop might span multiple windows, multiple candidate loops were always closer to each other in matrix and overlapped on fragments. We selected the candidate loops with the highest observed reads number for each overlapped entries cluster. Using this approach, the candidate loops might be affected by the linear distance between two genomic loci. In order to obtain the credible loops, we tested hypothesis that the number of reads observed at each entry $\mathbf{Mi}$j significantly enriched over the distance between each anchor of one loop. We calculated a p value for each candidate loop on the probability of observed reads number at Mij from a Poisson process with $\boldsymbol{\lambda=}\mathbf{|d}_{\mathbf{i-j}}\mathbf{|/}\mathbf{d}_{\mathbf{m}}$, in which $\mathbf{|d}_{\mathbf{i-j}}\mathbf{|}$ represented the distance between loop anchor and $\mathbf{d}_{\mathbf{m}}$ was the median length of candidate loops. We then used the standard Benjamini-Hochberg FDR control procedure to compute FDR and selected loops with FDR <= 0.05 as final chromatin loops.

**Overlaps between CSRs and loop**

We segmented genome by loop anchors and named these regions as loop anchor segmented regions. To compare the overlapped regions between CSRs and loop anchor segmented regions, we calculated the overlapped ratio by dividing the length of overlapped region to the length of merged regions between overlapped CSR and loop anchor segmented regions.

$$\mathbf{overlapped ratio=}\frac{\mathbf{length}\left( \boldsymbol{CSR\cap loop anchor segmented region} \right)}{\mathbf{length}\left( \boldsymbol{CSR\cup loop anchor segmented region} \right)}$$

CSRs with overlapped ratio greater than 0.6 were identified as overlapped with loop anchor regions.

**Simulation of reprogramming**

To capture the chromatin changes in different reprogramming processes, we compared fibroblast to NPC, cardiomyocyte and ESC to simulate the iN, iC and iPSC induction processes. We also compared the NPC and cardiomyocyte to hepatocytes to simulate the hepatic conversion with different initiated cell types. We compared compartments between two different cell types and captured regions switched from compartment B to A, with a genomic length longer than 40kb.

**Data availability**

All Hi-C, ATAC-seq, ChIP-seq and RNA-seq data generated in this study have been deposited to GEO with the accession number GSE148834 (GEO; GSE148834 access token: ujszuqsqjvapjyv).

Hi-C data, ATAC-seq, ChIP-seq and RNA-seq data download for analysis of simulating and characterizing iN, iC and iPSC processes and B-cell to Macrophage processes were from published data [21-32] and listed in Table S4.

**Code availability**

Code for Hi-C analysis is stored in website for Hi-C data analysis pipeline: <https://github.com/XuanCao-CX/HiCAT>. Code for call loops is stored in SigLoops (loop caller): <https://github.com/XuanCao-CX/SigLoops>.

Table S1

Table of Hi-C data

| **sample_name** | **raw data (reads number)** | **valid intra pairs (genomic distance >=500bp)** | **valid inter pairs** | **estimated resolution (kb）** |
| --- | --- | --- | --- | --- |
| HiC_0d_rep1 | 540,694,034 | 205,180,986 | 42,550,617 | 12.5 |
| HiC_0d_rep2 | 462,668,551 | 166,006,611 | 50,375,587 | 12.5 |
| HiC_2d_rep1 | 431,043,626 | 161,659,596 | 41,417,824 | 12.5 |
| HiC_2d_rep2 | 527,987,696 | 135,830,617 | 63,107,066 | 12.5 |
| HiC_4d_rep1 | 448,411,360 | 160,335,556 | 44,558,064 | 12.5 |
| HiC_4d_rep2 | 473,223,235 | 107,454,310 | 31,158,814 | 12.5 |
| HiC_6d_rep1 | 495,706,573 | 115,602,322 | 24,681,735 | 12.5 |
| HiC_6d_rep2 | 551,940,133 | 133,571,847 | 52,383,884 | 12.5 |
| HiC_14d_rep1 | 507,371,487 | 164,790,700 | 54,089,028 | 12.5 |
| HiC_14d_rep2 | 552,994,964 | 132,423,227 | 64,657,025 | 12.5 |

Table S2

Table of ATAC, RNA-seq and ChIP-seq data

| **sample_name** | **raw reads** | **mapped reads** |
| --- | --- | --- |
| ATAC_0d_1 | 81,881,205 | 59,206,483 |
| ATAC_0d_2 | 53,393,609 | 41,574,684 |
| ATAC_2d_1 | 104,825,384 | 53,283,559 |
| ATAC_2d_2 | 49,160,002 | 34,284,003 |
| ATAC_4d_1 | 70,801,240 | 53,273,274 |
| ATAC_4d_2 | 47,622,679 | 37,436,284 |
| ATAC_6d_1 | 28,700,149 | 21,051,905 |
| ATAC_6d_2 | 65,691,360 | 46,263,787 |
| ATAC_14d_1 | 24,521,390 | 19,275,561 |
| ATAC_14d_2 | 52,249,030 | 41,176,968 |
| TTF(RNA) | 16,513,445 | 15,422,227 |
| iHep_2d_rep1(RNA) | 11,331,696 | 10,496,870 |
| iHep_2d_rep2(RNA) | 13,490,730 | 12,505,879 |
| iHep_4d_rep1(RNA) | 12,170,738 | 11,303,638 |
| iHep_4d_rep2(RNA) | 13,472,725 | 12,531,428 |
| iHep_6d_rep1(RNA) | 16,382,514 | 15,241,510 |
| iHep_6d_rep2(RNA) | 12,416,268 | 11,557,607 |
| iHep_14d_rep1(RNA) | 15,864,932 | 14,807,610 |
| iHep_14d_rep2(RNA) | 11,920,845 | 11,098,745 |
| Liver(RNA) | 17,619,070 | 15,458,491 |
| H3K27ac_0d(ChIP) | 35,770,834 | 30,835,187 |
| H3K27ac_2d(ChIP) | 22,459,612 | 18,149,534 |
| H3K27ac_4d(ChIP) | 19,761,663 | 16,729,741 |
| H3K27ac_6d(ChIP) | 16,513,629 | 14,131,030 |
| H3K27ac_14d(ChIP) | 23,119,690 | 20,177,153 |
| H3K27ac_Hep(ChIP) | 19,619,674 | 17,361,205 |
| H3K27me3_0d(ChIP) | 21,013,110 | 16,418,845 |
| H3K27me3_2d(ChIP) | 18,439,873 | 14,282,585 |
| H3K27me3_4d(ChIP) | 18,250,012 | 13,874,704 |
| H3K27me3_6d(ChIP) | 62,385,027 | 43,546,627 |
| H3K27me3_14d(ChIP) | 23,197,789 | 15,201,938 |
| H3K27me3_Hep(ChIP) | 20,282,308 | 15,680,264 |
| Foxa3_0d(ChIP) | 21,001,138 | 16,451,291 |
| Foxa3_2d(ChIP) | 20,098,668 | 16,779,263 |
| Foxa3_4d(ChIP) | 20,738,096 | 16,371,801 |
| Foxa3_6d(ChIP) | 21,687,065 | 16,257,423 |
| Foxa3_14d(ChIP) | 24,368,015 | 17,947,374 |
| Gata4_2d(ChIP) | 26,712,049 | 20,924,390 |
| Hnf1a_2d(ChIP) | 27,594,853 | 19,066,745 |
| CTCF_0d(ChIP) | 24,838,915 | 19,201,260 |
| CTCF_2d(ChIP) | 40,748,470 | 20,526,003 |
| CTCF_4d(ChIP) | 44,194,887 | 15,815,059 |
| CTCF_6d(ChIP) | 26,526,305 | 19,366,414 |
| CTCF_14d(ChIP) | 23,335,112 | 17,955,101 |
| H3K4me1_0d(ChIP) | 28,805,810 | 25,703,173 |
| H3K4me1_2d(ChIP) | 28,001,613 | 25,524,242 |
| H3K4me1_4d(ChIP) | 24,957,409 | 22,324,299 |
| H3K4me1_6d(ChIP) | 23,767,268 | 21,138,411 |
| H3K4me1_14d(ChIP) | 25,106,940 | 21,081,021 |
| H3K4me1_Hep(ChIP) | 16,960,109 | 14,814,851 |
| H3K4me3_0d(ChIP) | 20,785,015 | 17,932,396 |
| H3K4me3_2d(ChIP) | 19,264,356 | 16,806,393 |
| H3K4me3_4d(ChIP) | 16,670,303 | 14,700,776 |
| H3K4me3_6d(ChIP) | 4,080,417 | 3,289,179 |
| H3K4me3_14d(ChIP) | 20,358,415 | 18,409,096 |
| H3K4me3_Hep(ChIP) | 18,097,924 | 14,289,539 |

Table S3

Table of primer

| **name** | **sequence** |
| --- | --- |
| Linear_verctor_forawd_primer | tagctagctagtcgagctcaacttcgaattcgatat |
| Linear_verctor_reverse_primer | ggtattatcatcgtgtttttcaaaggaaaaccacgtcc |
|  |  |
| mCherry_forward_primer | acacgatgataataccatggtgagcaagggcgagga |
| mCHerry_reverse_primer | tcgactagctagctacttgtacagctcgtccatgc |
|  |  |
| zSGreen_forward_primer | gaaaaacacgatgataataccatggcccagtccaagcacgg |
| zSGreen_reverse_primer | agctcgactagctagttagggcaaggcggagccggagg |
|  |  |
| HSA_forward_primer | tgaaaaacacgatgataataccatgggcagagcgatggtg |
| HSA_reverse_primer | gttgagctcgactagctagctaacagtagagatgtagaagag |

Table S4

Table of antibody in this study

| **Antibody** | **Concentration** | **Experiment** | **Source** | **Cat#** |
| --- | --- | --- | --- | --- |
| E-cadherin | 1:1000 | IF | GeneTex | GTX100443 |
| Hnf4a | 1:1000 | IF | Abcam | ab181604 |
| DAPI | 1:500 | IF | Roche | 10236276001 |
| Cy™3 AffiniPure Donkey Anti-Rabbit IgG (H+L) | 1:500 | IF | Jackson ImmunoResearch | 711-165-152 |
| Foxa3 | 5μg | ChIP | Santa Cruz | sc-5361 |
| Gata4 | 5μg | ChIP | Santa Cruz | sc-1237 |
| Hnf1a | 5μg | ChIP | Santa Cruz | sc-6547 |
| CTCF | 5μg | ChIP | Active Motif | 61312 |
| H3K27ac | 5μg | ChIP | Abcam | ab4729 |
| H3K27me3 | 5μg | ChIP | CST | 9733 |

Table S5

Table of public data reanalyzed in this study

| Cell_type | Data_type | Liver | reference |
| --- | --- | --- | --- |
| Cardiomyocyte | H3K27ac | GSE73769 | PMID: 28394251 [28] |
| Cardiomyocyte | H3K27me3 | GSE73769 |  |
| NPC | RNA | GSE96107 | PMID: 29053968 [26] |
| NPC | ATAC | GSE96107 |  |
| NPC | H3K27ac | GSE96107 |  |
| NPC | H3K27me3 | GSE96107 |  |
| NPC | Hi-C | GSE96107 |  |
| NPC | CTCF | GSE96107 |  |
| ESC | RNA | GSM2533844 |  |
| ESC | Hi-C | GSE96107 |  |
| ESC | CTCF | GSE96107 |  |
| MEF | ATAC | GSE90892 | PMID: 28111071 [23] |
| MEF | H3K27ac | GSE90893 |  |
| MEF | H3K27me3 | GSE90893 |  |
| ESC | ATAC | GSE90892 |  |
| ESC | H3K27ac | GSE90893 |  |
| ESC | H3K27me3 | GSE90893 |  |
| ESC | Oct4 ChIPseq | GSE90893 |  |
| B-cells | RNA | GSM4183318, GSM4183328 | PMID: 32514124 [32] |
| B-cells | H3K27ac | ERS4536024, ERS4536025 |  |
| B-cells | Hi-C | GSM4198745, GSM4198755 |  |
| iMac | RNA | GSM4183327, GSM4183337 |  |
| iMac | ATAC | GSM4224778, GSM4224779 |  |
| iMac | H3K27ac | ERS4536012, ERS4536013 |  |
| iMac | Hi-C | GSM4198754, GSM4198764 |  |
| Liver | CTCF | GSE91731 | PMID: 22955616 [24] |
| Cardiomyocyte | Tbx5 ChIPseq | GSE21529 | PMID: 21415370 [27] |
| MEF | Hi-C | GSE76479 | PMID: 26971819 [21] |
| Hep | ATAC | GSE111499 | PMID: 31271748 [22] |
| Cardiomyocyte | ATAC | GSE123457 | PMID: 30773489 [30] |
| Cardiomyocyte | RNA | GSE96690 | PMID: 28802249 [33] |
| Cardiomyocyte | Hi-C | GSE96692 |  |
| Cardiomyocyte | CTCF | GSE96691 |  |
| MEF | CTCF | GSE36027 | PMID: 22763441 [25] |
| B-cells | ATAC | GSM3791272, GSM3791273 | PMID: 32514124 [32] |
| Liver | Hi-C | GSE65126 | PMID: 25732821 [34] |
| NPC | Ascl1 ChIPseq | GSE48336 | PMID: 23891001 [29] |

**Supplemental Figure Legends**

**Fig. S1. Efficient hepatic conversion from fibroblasts.**

(**A**) Foxa3 and mCherry were co-expressed by constructing in the same plasmid. Gata4 and zSGreen, and Hnf1a and HSA were also co-expressed. Histogram showed the proportion of cells expressing mCherry, zSGreen and HSA simultaneously. Data presented as mean + s.d. (**B**) Epithelial conversion from fibroblasts to iHep was confirmed by immunofluorescent staining of E-cadherin (red membrane staining). Foxa3 and GFP are co-expressed by constructing in the same plasmid. Nuclei are stained blue by DAPI. Histogram shows proportion of cells expressing both E-cadherin and Foxa3. (**C**) Hepatic identity establishment of iHep was confirmed by immunofluorescent staining of hepatic transcription factor Hnf4a (red nuclei staining). Foxa3 and GFP were co-expressed by constructing in the same plasmid. Nuclei were stained blue by DAPI. Histogram showed proportion of cells expressing Hnf4a and Foxa3 simultaneously.

**Fig. S2. Global changes in transcriptome and chromatin status during hepatic conversion.**

(**A**) Scatter plot of PC1, PC2 and PC3 values of principal component analysis (PCA) on gene expression levels of all time points during hepatic conversion. Transcriptome changed towards hepatocyte like but were not identical to hepatocyte at day 14. (**B**) Boxplots of gene expression for up-regulated genes during hepatic conversion and associated gene ontology enrichment analysis by Metascape. Top 10 pathways were shown. (**C**) Reviewer Figure 4. Venn diagram showed the comparison between CSRs during iHep induction (orange) and chromatin regions showing different compartments between fibroblasts and hepatocytes (green). The numbers of CSRs or different compartment regions were shown. Boxplots of gene expression for down-regulated genes during hepatic conversion and associated gene ontology enrichment analysis by Metascape. (**D**) Scatter plot of PC1, PC2 and PC3 values of PCA on H3K27ac levels, H3K27me3 levels and ATAC signals at different time points during hepatic conversion. H3K27ac, H3K27me3 and ATAC signal changed towards hepatocytes at day 14. (**E**) Boxplots of H3K27ac levels, H3K27me3 levels and ATAC signals at up-regulated genes during hepatic conversion. Boxplots of H3K27me3 at genes with or without H3K27me3 mark were shown separately. (**F**) Scatter plots showed the correlation between the fold change of H3K27ac level, H3K27me3 level, ATAC signal and fold change of genes expression in iHep induction. Pearson correlation coefficients are shown in red at the corner of each graph. (**G**) Representative examples of genomic regions around activated hepatic genes, *Hnf4a* and *Trf.* ATAC signal (top, violet), H3K27ac (middle, pink), H3K27me3 (bottom, purple) and gene expression (bottom, orange) were shown. The genomic regions covering the gene body of *Hnf4a* and *Trf* were highlighted by grey shadow.

**Fig. S3. Global changes in compartmentalization during hepatic conversion.**

(**A**) Scatter plot of PC1, PC2 and PC3 values of PCA on compartmentalization during hepatic conversion. (**B**) Percentage of the length of regions showing compartment B-to-A and A-to-B switch. (**C**) Venn diagram showed the number of regions in CSRs during iHep induction (orange), chromatin regions showing different compartments between fibroblasts and hepatocytes (green) and their overlapped regions (number of regions in CSRs). (**D**) Pie graph showing the number and percentage of B-to-A, A-to-B and transient CSR. (**E**) A representative example of the dynamic compartmentalization during hepatic conversion. Compartment was shown by PC1 track at 40kb resolution. Red shadows and blue shadows highlight regions with B-to-A and A-to-B compartment changes, respectively. (**F**, **G**) Differential Hi-C heatmap showed alteration of chromatin interaction of fibroblast and iHep cells at chr2:69,000,000-89,000,000 (F) and chr3:36,000,000-56,000,000 (G) at resolution of 40kb, which was determined by subtraction of contact matrix of fibroblast and day 14 iHep cells. Dashed box highlighted interaction change between compartment switchable regions and unswitched compartment A or B.

**Fig. S4. Hepatic genes expressed in B-to-A CSRs.**

(**A**) Heatmap showing gene expression in B-to-A CSRs. Functional and regulatory hepatic genes were found in cluster of induced genes. Example gene names were listed next to heatmap. (**B**) Boxplots of expression levels of genes located in A-to-B CSRs. Wilcoxon rank sum test was applied. (**C**) Gene ontology enrichment analysis for downregulated genes in A-to-B CSRs by Metascape. We also performed gene ontology enrichment analysis on downregulated genes in A-to-B CSRs by GSEA, with no significant fibroblast associated pathway being reported. (**D**) Heatmap showing gene expression in A-to-B CSRs. Example of fibroblast associated genes were listed next to the heatmap.

**Fig. S5. Chromatin structure changes in TADs and loops.**

(**A**) Boxplot showing the average genomic length of TADs and loops. (**B**) Representative examples of showing the compartmentalization, TADs and related chromatin interactions. Heatmaps were plotted at resolution of 40kb. (**C**) Distribution of CTCF around loop anchors (anchors±100kb). (**D**) Heatmap showed the average chromatin interaction around loop anchors (±20kb) at loop interaction increased loop and interaction decreased loops.

**Fig. S6. Chromatin status change in A-to-B CSRs.**

(**A**) Three patterns of compartment switch in A-to-B CSRs. Vertical dashed lines indicate CSR boundaries. (**B**) K-means clustering was performed on the PC1 values at 40kb bin size in vanished, emerged and shifted A-to-B CSRs during hepatic conversion. (**C**, **D**) Compartmentalization corresponding to vanished, emerged and shifted A-to-B CSRs and flanking regions. PC1 values were used to profile compartmentalization. Vertical dashed lines indicate the boundary of CSRs. The horizontal dotted lines indicate the threshold of compartment switch (PC1=0) (**C**). Broken line graph of the sum of PC1 values in vanished, emerged and shifted A-to-B CSRs during hepatic conversion. Data were presented as mean ± s.d (**D**). (**E**, **F**) ATAC signal corresponding to vanished, emerged and shifted A-to-B CSRs and flanking regions (E). Boxplots showing the kinetics of ATAC signal at individual peak in vanished, emerged and shifted A-to-B CSRs (F).

**Fig. S7. Loss of Brg1 decreased the efficiency of iHep induction.**

(**A**) Heatmap showing the Brg1 binding at all ATAC peaks in compartment B for wild-type (WT) fibroblasts, and 5kb around the peak center was shown. (**B**) Heatmap showing the chromatin accessibility at all ATAC peaks in compartment B for WT and Brg1-repressed cells, and 5kb around the peak center was shown. Data were retrieved from [35]. (**C**) Distribution of chromatin accessibility around ATAC peaks in compartment B for Snf2h-repressed cells. (**D**) QPCR showing the decrease of Brg1 in mRNA level after Brg1 knock-down. (**E**) Histogram showing the clone number of iHep which is induced from WT and Brg1 repressed fibroblast. (**F**) Histogram showing the clone number of iHep which is induced from fibroblast at different PFI-3 concentration. (**G**) Distribution of chromatin accessibility around ATAC peaks in compartment B for Snf2h-repressed cells, and 5kb around the peak center was shown. Data were retrieved from [35]. (**H**) Histogram showing the clone number of iHep which is induced from WT and Snf2h repressed fibroblast.

**Fig. S8. Histone modification changes in CSRs.**

(**A, B**) H3K27ac modification corresponding to vanished, emerged and shifted B-to-A CSRs and flanking regions (A). Broken line graph showing the kinetics of H3K27ac level in vanished, emerged and shifted B-to-A CSRs during hepatic conversion (B). (**C**, **D**) H3K27me3 modification corresponding to vanished, emerged and shifted B-to-A CSRs and flanking regions (C). Broken line graph showing the kinetics of H3K27me3 level in vanished, emerged and shifted B-to-A CSRs (D). (**E**) Heatmap showing the H3K27ac, H3K27me3, H3K4me1 and H3K4me3 modification kinetics at all four clusters ATAC peaks in Fig. 2I. (**F, G**) H3K27ac modification corresponding to vanished, emerged and shifted A-to-B CSRs and flanking regions (F). Broken line graph showing the kinetics of H3K27ac level in vanished, emerged and shifted A-to-B CSRs during hepatic conversion (G). (**H**, **I**) H3K27me3 modification corresponding to vanished, emerged and shifted A-to-B CSRs and flanking regions (H). Broken line graph showing the kinetics of H3K27me3 level in vanished, emerged and shifted A-to-B CSRs (I).

**Fig. S9. CTCF binding was adjacent to loop anchors.**

(**A**) A representative example during hepatic conversion, showing CTCF binding in the genomic region. (**B**) Heatmap showing all CTCF binding during hepatic conversion.

**Fig. S10. Foxa3 binding in B-to-A CSRs.**

(**A**) Heatmaps showing the Foxa3 (green), Gata4 (blue) and Hnf1a (orange) binding at day 2. (**B**) A representative genomic region showing the compartmentalization (top) and Foxa3 binding (middle, green), Gata4 binding (middle, blue) and Hnf1a binding (bottom, orange) at day 2. (**C**, **D**) Foxa3 binding level corresponding to vanished, emerged and shifted A-to-B CSRs and flanking regions. Vertical dashed lines indicate CSR boundaries (C). Broken line graph showing the kinetics of Foxa3 binding in vanished, emerged and shifted A-to-B CSRs. Data were presented as mean ± s.d (D).

**Fig. S11. Foxa3 binds chromatin accessible sites in B-to-A CSRs.**

(**A**) Heatmap of ATAC signals and Foxa3 binding at pre-existing chromatin accessible sites in B-to-A CSRs. Heatmap showed the signals at ATAC summit ±5kb, and these regions were ranked by decreased ATAC signal. (**B**) Heatmap showing the kinetics of Foxa3 binding at ATAC peaks of all four clusters in Fig, 2I. (**C**) Broken line graph showing the binding of Foxa3 (shown as rpm) to B-to-A CSRs in B-to-A CSRs with or without induced genes. (**D**) Representative examples of hepatic genes, *Arhgef28*, in B-to-A CSRs, showing the compartmentalization (top), Foxa3 binding (middle, green), ATAC signal (middle, violet), H3K27ac (middle, pink), H3K27me3 (middle, purple) distribution and gene expression (bottom, orange) during hepatic conversion. Gene locations were indicated by arrows. (**E**) Representative examples of chromatin interactions around hepatic gene *Arhgef28*, at 40kb resolution*.* Dotted line highlighted chromatin interaction changes at *Arhgef28* associated loop anchor.

**Fig. S12. Mosaic status in compartment B for cell identity conversion from fibroblasts to NPCs and cardiomyocytes.**

(**A-H**) Fibroblasts and NPCs were compared to analyze mosaic status in switchable compartment B. Genomic length of B-to-A CSRs and unswitched compartment regions (B). Gene density of B-to-A CSRs and unswitched B regions (C). Distribution of CTCF binding at boundaries of B-to-A CSRs. Boundary ±100kb was shown (D). Boxplots of average ATAC signal (E) and Ascl1 binding level (F) in B-to-A CSRs (orange) and unswitched compartment B regions (blue). Wilcoxon rank sum test was applied. Boxplots showing average H3K27ac (G) and H3K27me3 (H) in B-to-A CSRs. (**I-P**) Fibroblasts and cardiomyocytes were compared to analyze mosaic status in switchable compartment B (I). Genomic length of B-to-A CSRs and unswitched compartment regions (J). Gene density of B-to-A CSRs and unswitched B regions (K). Distribution of CTCF binding at boundaries of B-to-A CSRs. Boundary ±100kb was shown (L). Boxplots of average ATAC signal (M) and Tbx5 binding level (N) in B-to-A CSRs (orange) and unswitched compartment B regions (blue). Wilcoxon rank sum test was applied. Boxplots showing average H3K27ac (O) and H3K27me3 (P) in B-to-A CSRs.

**Fig. S13. Mosaic status at induced genes from B-cells to macrophages.**

(**A**) Schematic overview of transdifferentiation from B cells to induced macrophages. (**B**) Distribution of CTCF binding at boundaries of B-to-A CSRs. Boundary ±100kb was shown. (**C**, **D**) Boxplots of average ATAC signals (C) and CEBPA binding levels (D) in B-to-A CSRs (orange) and unswitched compartment B regions (blue). Wilcoxon rank sum test was applied. (**E**, **F**) Boxplots showing average H3K27ac (E) and gene expression (F) in B-to-A CSRs.

Reference

1. Huang P, He Z, Ji S, et al. Induction of functional hepatocyte-like cells from mouse fibroblasts by defined factors. *Nature* 2011; **475**: 386-9.

2. Buenrostro JD, Wu B, Chang HY, et al. ATAC-seq: A Method for Assaying Chromatin Accessibility Genome-Wide. *Curr Protoc Mol Biol* 2015; **109**: 21 9 1- 9 9.

3. Tarlow BD, Finegold MJ, Grompe M. Clonal tracing of Sox9+ liver progenitors in mouse oval cell injury. *Hepatology* 2014; **60**: 278-89.

4. Kim D, Pertea G, Trapnell C, et al. TopHat2: accurate alignment of transcriptomes in the presence of insertions, deletions and gene fusions. *Genome Biol* 2013; **14**: R36.

5. Trapnell C, Roberts A, Goff L, et al. Differential gene and transcript expression analysis of RNA-seq experiments with TopHat and Cufflinks. *Nat Protoc* 2012; **7**: 562-78.

6. Anders S, Pyl PT, Huber W. HTSeq--a Python framework to work with high-throughput sequencing data. *Bioinformatics* 2015; **31**: 166-9.

7. Love MI, Huber W, Anders S. Moderated estimation of fold change and dispersion for RNA-seq data with DESeq2. *Genome Biol* 2014; **15**: 550.

8. Zhou Y, Zhou B, Pache L, et al. Metascape provides a biologist-oriented resource for the analysis of systems-level datasets. *Nat Commun* 2019; **10**: 1523.

9. Martin M. Cutadapt Removes Adapter Sequences From High-Throughput Sequencing Reads. *EMBnetjournal* 2011; **17**.

10. Langmead B, Trapnell C, Pop M, et al. Ultrafast and memory-efficient alignment of short DNA sequences to the human genome. *Genome Biol* 2009; **10**: R25.

11. Zhang Y, Liu T, Meyer CA, et al. Model-based analysis of ChIP-Seq (MACS). *Genome Biol* 2008; **9**: R137.

12. Pinello L, Xu J, Orkin SH, et al. Analysis of chromatin-state plasticity identifies cell-type-specific regulators of H3K27me3 patterns. *Proc Natl Acad Sci U S A* 2014; **111**: E344-53.

13. Thorvaldsdottir H, Robinson JT, Mesirov JP. Integrative Genomics Viewer (IGV): high-performance genomics data visualization and exploration. *Brief Bioinform* 2013; **14**: 178-92.

14. James T. Robinson HT, Wendy Winckler, Mitchell Guttman, Eric S. Lander, Gad Getz, Jill P. Mesirov. Integrative Genomics Viewer. *Nat Biotechnol* 2011; **29**: 24-6.

15. Reske JJ, Wilson MR, Chandler RL. ATAC-seq normalization method can significantly affect differential accessibility analysis and interpretation. *Epigenetics Chromatin* 2020; **13**: 22.

16. Rao SS, Huntley MH, Durand NC, et al. A 3D map of the human genome at kilobase resolution reveals principles of chromatin looping. *Cell* 2014; **159**: 1665-80.

17. Lieberman-Aiden E, van Berkum NL, Williams L, et al. Comprehensive mapping of long-range interactions reveals folding principles of the human genome. *Science* 2009; **326**: 289-93.

18. Shin H, Shi Y, Dai C, et al. TopDom: an efficient and deterministic method for identifying topological domains in genomes. *Nucleic Acids Res* 2016; **44**: e70.

19. Zhou X, Maricque B, Xie M, et al. The Human Epigenome Browser at Washington University. *Nat Methods* 2011; **8**: 989-90.

20. Crane E, Bian Q, McCord RP, et al. Condensin-driven remodelling of X chromosome topology during dosage compensation. *Nature* 2015; **523**: 240-4.

21. Krijger PH, Di Stefano B, de Wit E, et al. Cell-of-Origin-Specific 3D Genome Structure Acquired during Somatic Cell Reprogramming. *Cell Stem Cell* 2016; **18**: 597-610.

22. Li W, Yang L, He Q, et al. A Homeostatic Arid1a-Dependent Permissive Chromatin State Licenses Hepatocyte Responsiveness to Liver-Injury-Associated YAP Signaling. *Cell Stem Cell* 2019; **25**: 54-68 e5.

23. Chronis C, Fiziev P, Papp B, et al. Cooperative Binding of Transcription Factors Orchestrates Reprogramming. *Cell* 2017; **168**: 442-59 e20.

24. Consortium EP. An integrated encyclopedia of DNA elements in the human genome. *Nature* 2012; **489**: 57-74.

25. Shen Y, Yue F, McCleary DF, et al. A map of the cis-regulatory sequences in the mouse genome. *Nature* 2012; **488**: 116-20.

26. Bonev B, Mendelson Cohen N, Szabo Q, et al. Multiscale 3D Genome Rewiring during Mouse Neural Development. *Cell* 2017; **171**: 557-72 e24.

27. He A, Kong SW, Ma Q, et al. Co-occupancy by multiple cardiac transcription factors identifies transcriptional enhancers active in heart. *Proc Natl Acad Sci U S A* 2011; **108**: 5632-7.

28. Ai S, Peng Y, Li C, et al. EED orchestration of heart maturation through interaction with HDACs is H3K27me3-independent. *Elife* 2017; **6**.

29. Webb AE, Pollina EA, Vierbuchen T, et al. FOXO3 shares common targets with ASCL1 genome-wide and inhibits ASCL1-dependent neurogenesis. *Cell Rep* 2013; **4**: 477-91.

30. Monroe TO, Hill MC, Morikawa Y, et al. YAP Partially Reprograms Chromatin Accessibility to Directly Induce Adult Cardiogenesis In Vivo. *Dev Cell* 2019; **48**: 765-79 e7.

31. Rosa-Garrido M, Chapski DJ, Schmitt AD, et al. High-Resolution Mapping of Chromatin Conformation in Cardiac Myocytes Reveals Structural Remodeling of the Epigenome in Heart Failure. *Circulation* 2017; **136**: 1613-25.

32. Stik G, Vidal E, Barrero M, et al. CTCF is dispensable for immune cell transdifferentiation but facilitates an acute inflammatory response. *Nat Genet* 2020; **52**: 655-61.

33. Rosa-Garrido M, Chapski DJ, Schmitt AD, et al. High-Resolution Mapping of Chromatin Conformation in Cardiac Myocytes Reveals Structural Remodeling of the Epigenome in Heart Failure. *Circulation* 2017; **136**: 1613-25.

34. Vietri Rudan M, Barrington C, Henderson S, et al. Comparative Hi-C reveals that CTCF underlies evolution of chromosomal domain architecture. *Cell Rep* 2015; **10**: 1297-309.

35. Jegu T, Blum R, Cochrane JC, et al. Xist RNA antagonizes the SWI/SNF chromatin remodeler BRG1 on the inactive X chromosome. *Nat Struct Mol Biol* 2019; **26**: 96-109.
