# Supplementary material for: Pre-existing chromatin accessibility of switchable repressive compartment delineates cell plasticity
Source: Natl Sci Rev. 2021 Dec 31;9(6):nwab230. doi: 10.1093/nsr/nwab230 (PMC9249582; doi:10.1093/nsr/nwab230)
Supplement: nwab230_Supplemental_Files [file nwab230_supplemental_files.zip › Supplymentary_Figure_NSR_202110.pdf]

Figure S1

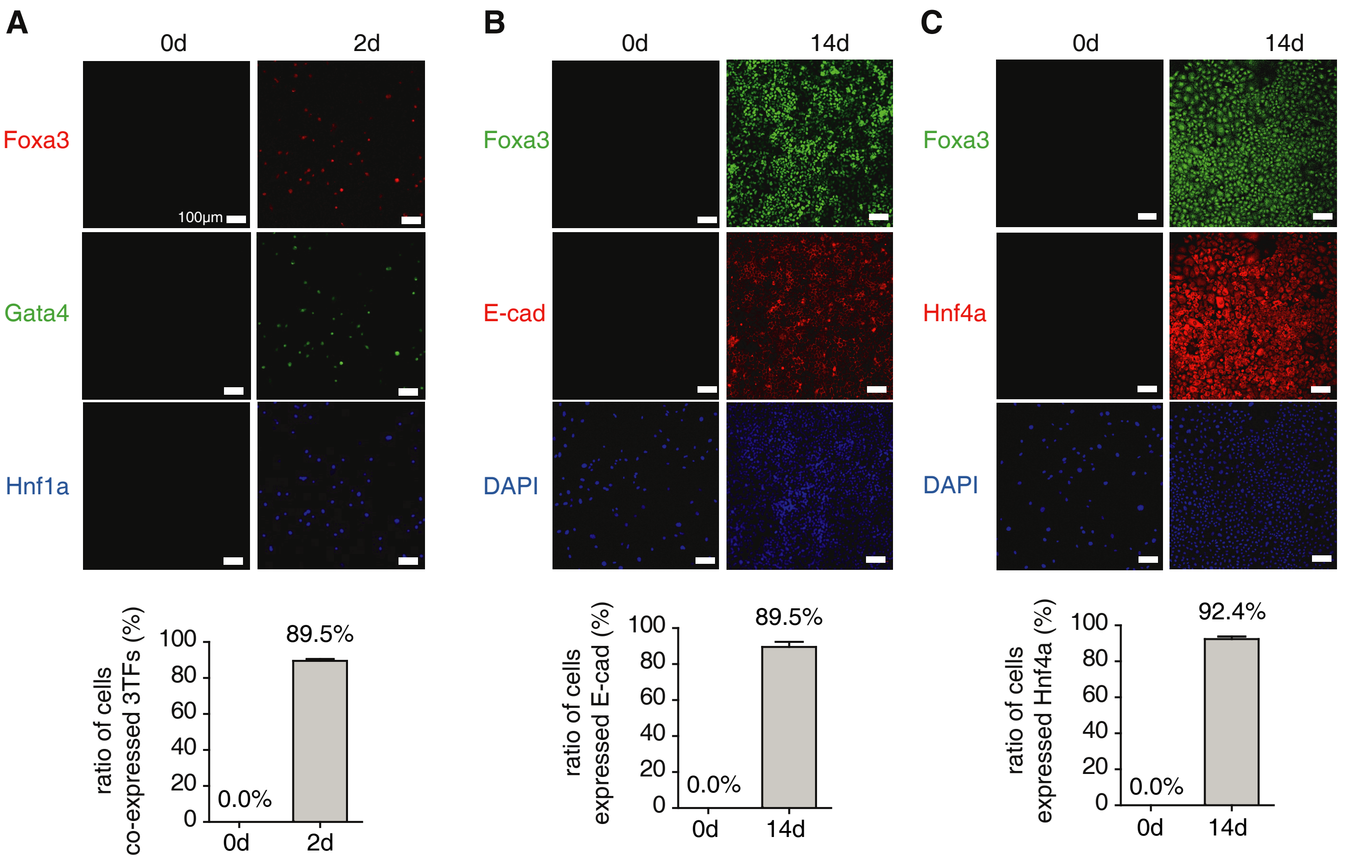

A
0d
2d
Foxa3
100µm
Gata4
Hnf1a
ratio of cells co-expressed 3TFs (%)
100
80
60
40
20
0
0.0%
89.5%
0d
2d
B
0d
14d
Foxa3
E-cad
DAPI
ratio of cells expressed E-cad (%)
100
80
60
40
20
0
0.0%
89.5%
0d
14d
C
0d
14d
Foxa3
Hnf4a
DAPI
ratio of cells expressed Hnf4a (%)
100
80
60
40
20
0
0.0%
92.4%
0d
14d

Figure S2

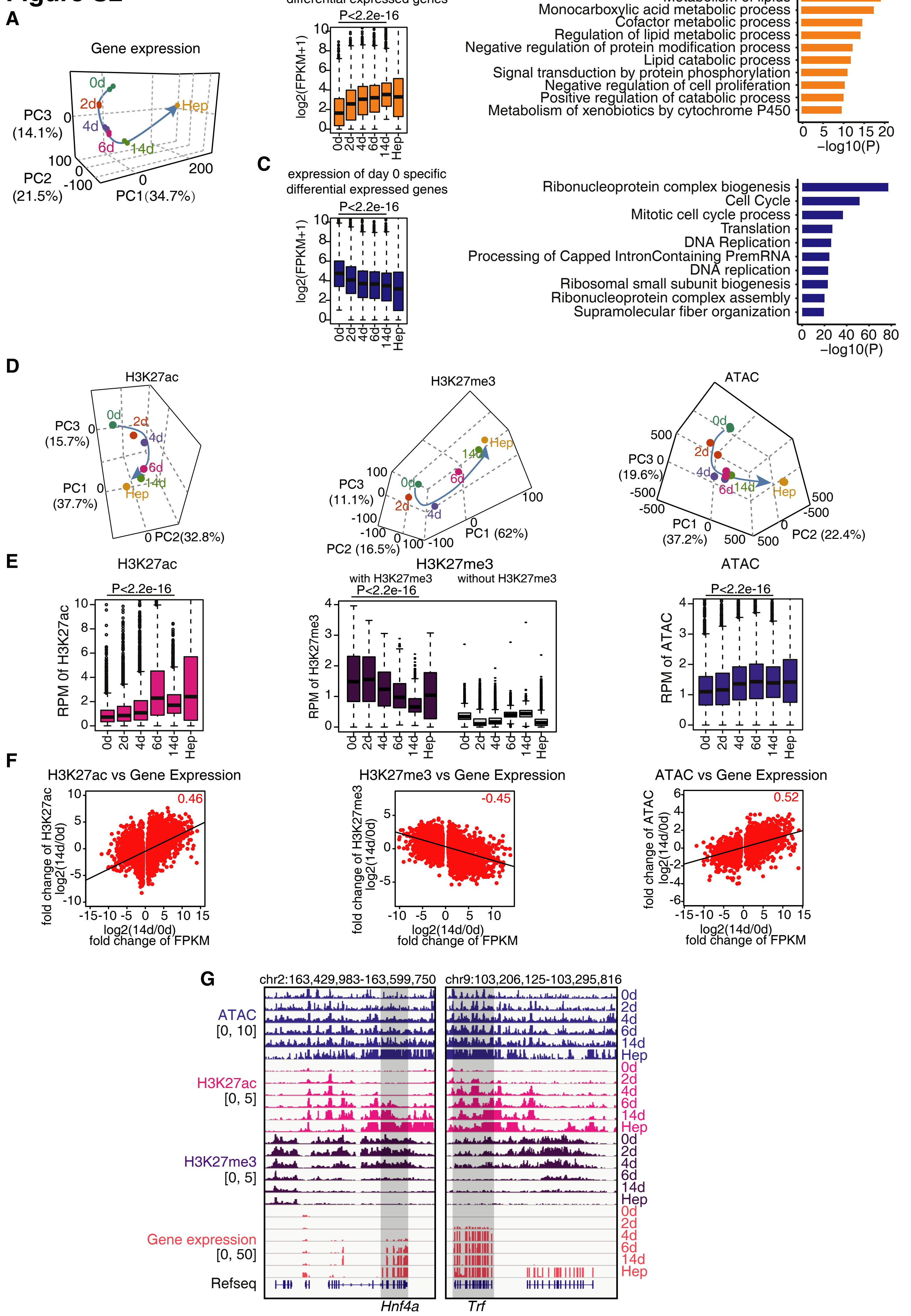
A
Gene expression
PC3 (14.1%)
PC2 (21.5%)
PC1 (34.7%)
0d
2d
4d
6d
14d
Hep
B
expression of day 14 specific differential expressed genes
P<2.2e-16
log2(FPKM+1)
Metabolism of lipids
Monocarboxylic acid metabolic process
Cofactor metabolic process
Regulation of lipid metabolic process
Negative regulation of protein modification process
Lipid catabolic process
Signal transduction by protein phosphorylation
Negative regulation of cell proliferation
Positive regulation of catabolic process
Metabolism of xenobiotics by cytochrome P450
−log10(P)
C
expression of day 0 specific differential expressed genes
P<2.2e-16
log2(FPKM+1)
Ribonucleoprotein complex biogenesis
Cell Cycle
Mitotic cell cycle process
Translation
DNA Replication
Processing of Capped IntronContaining PremRNA
DNA replication
Ribosomal small subunit biogenesis
Ribonucleoprotein complex assembly
Supramolecular fiber organization
−log10(P)
D
H3K27ac
PC3 (15.7%)
PC1 (37.7%)
PC2(32.8%)
H3K27me3
PC3 (11.1%)
PC2 (16.5%)
PC1 (62%)
ATAC
PC3 (19.6%)
PC1 (37.2%)
PC2 (22.4%)
E
H3K27ac
P<2.2e-16
RPM 0f H3K27ac
H3K27me3
with H3K27me3
without H3K27me3
P<2.2e-16
RPM of H3K27me3
ATAC
P<2.2e-16
RPM of ATAC
F
H3K27ac vs Gene Expression
0.46
fold change of H3K27ac log2(14d/0d)
log2(14d/0d) fold change of FPKM
H3K27me3 vs Gene Expression
-0.45
fold change of H3K27me3 log2(14d/0d)
log2(14d/0d) fold change of FPKM
ATAC vs Gene Expression
0.52
fold change of ATAC log2(14d/0d)
log2(14d/0d) fold change of FPKM
G
chr2:163,429,983-163,599,750
chr9:103,206,125-103,295,816
ATAC [0, 10]
H3K27ac [0, 5]
H3K27me3 [0, 5]
Gene expression [0, 50]
Refseq
Hnf4a
Trf

Figure S3

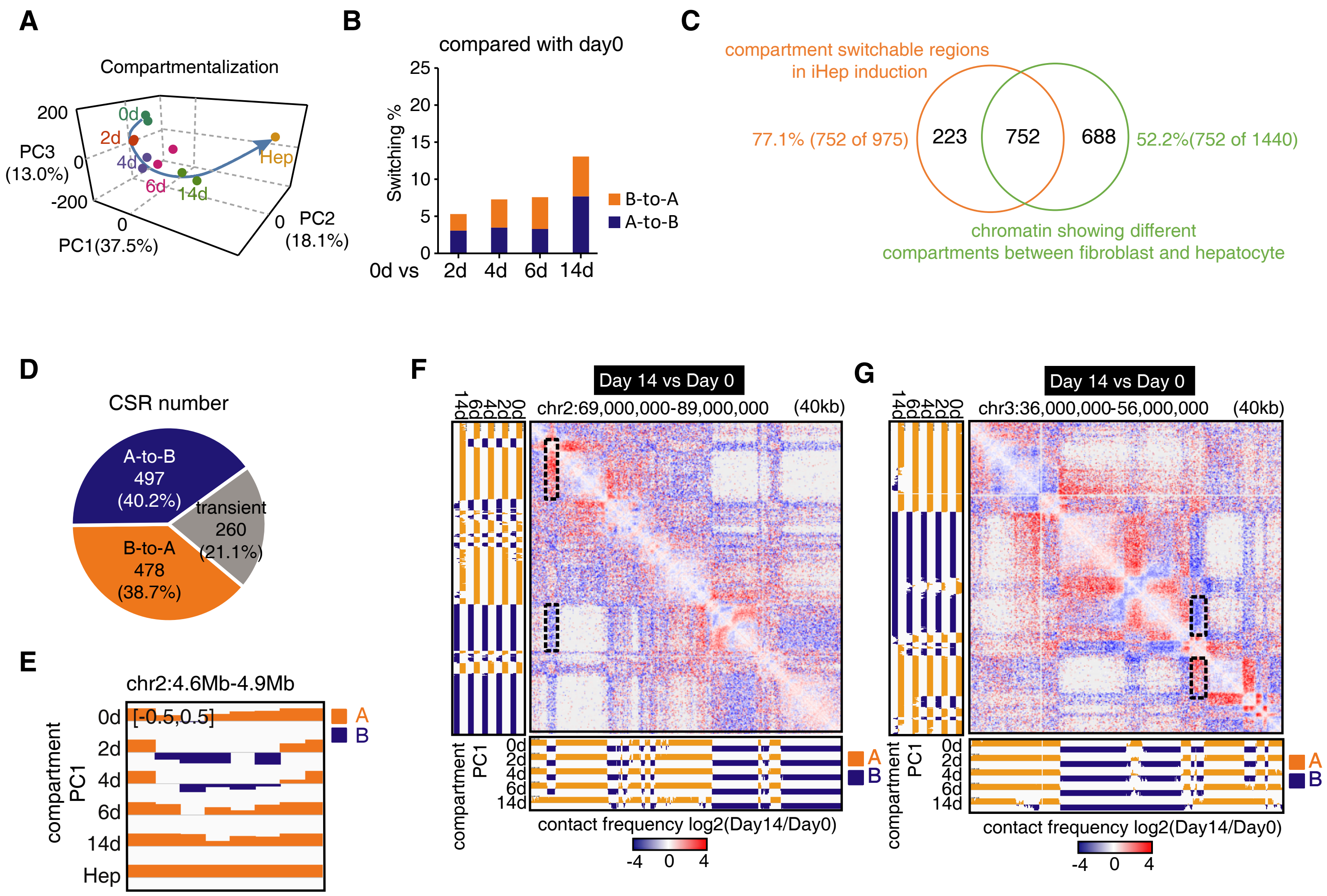
A
Compartmentalization
PC3 (13.0%)
PC1(37.5%)
PC2 (18.1%)
0d
2d
4d
6d
14d
Hep
B
compared with day0
Switching %
B-to-A
A-to-B
0d vs
2d
4d
6d
14d
C
compartment switchable regions in iHep induction
77.1% (752 of 975)
223
752
688
52.2%(752 of 1440)
chromatin showing different compartments between fibroblast and hepatocyte
D
CSR number
A-to-B 497 (40.2%)
transient 260 (21.1%)
B-to-A 478 (38.7%)
E
chr2:4.6Mb-4.9Mb
[-0.5,0.5]
compartment PC1
A
B
F
Day 14 vs Day 0
chr2:69,000,000-89,000,000
(40kb)
contact frequency log2(Day14/Day0)
G
Day 14 vs Day 0
chr3:36,000,000-56,000,000
(40kb)
contact frequency log2(Day14/Day0)

Figure S4

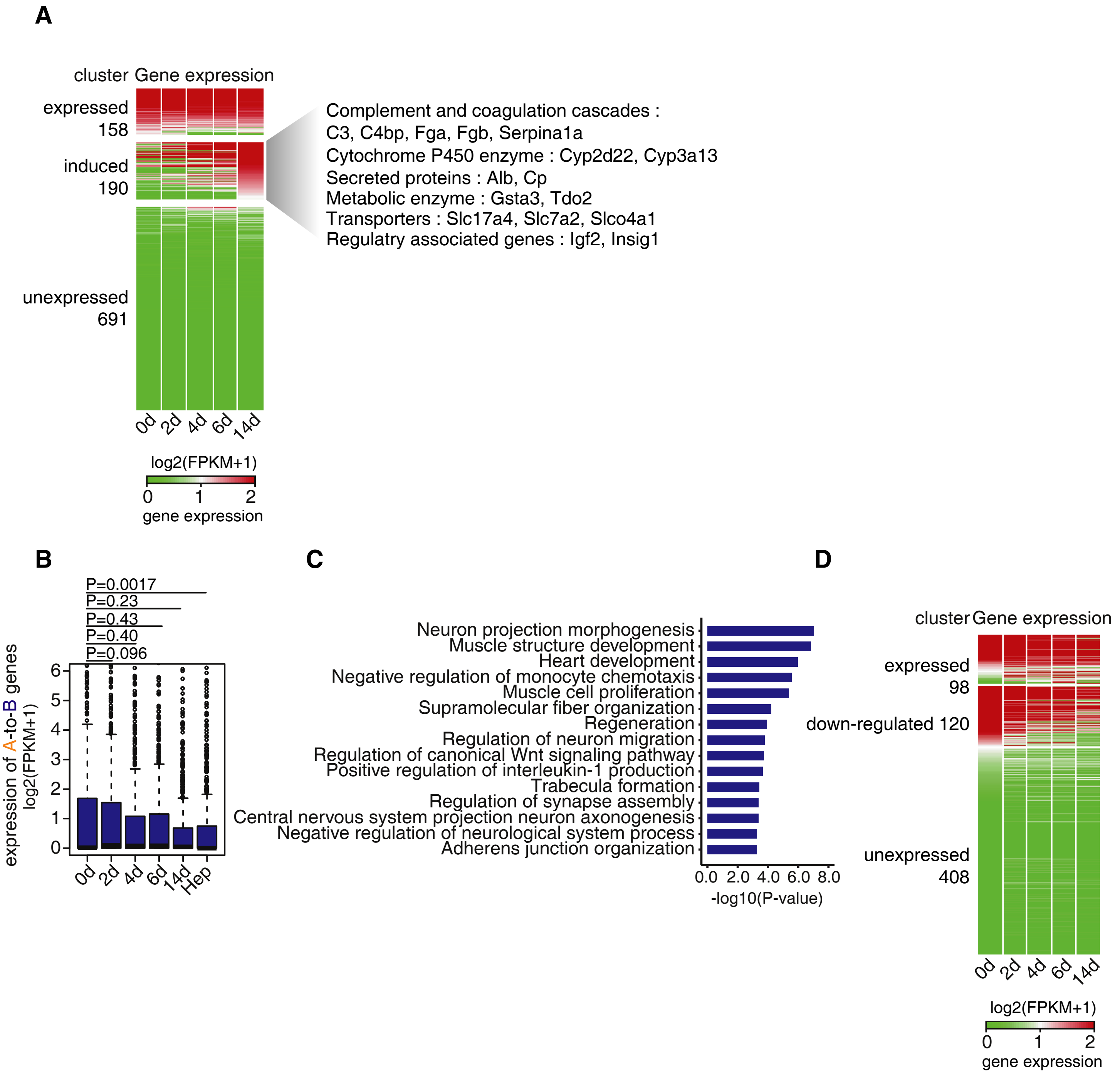
A
cluster Gene expression
expressed 158
induced 190
unexpressed 691
0d 2d 4d 6d 14d
log2(FPKM+1)
0 1 2
gene expression
Complement and coagulation cascades :
C3, C4bp, Fga, Fgb, Serpina1a
Cytochrome P450 enzyme : Cyp2d22, Cyp3a13
Secreted proteins : Alb, Cp
Metabolic enzyme : Gsta3, Tdo2
Transporters : Slc17a4, Slc7a2, Slco4a1
Regulatry associated genes : Igf2, Insig1
B
P=0.0017
P=0.23
P=0.43
P=0.40
P=0.096
expression of A-to-B genes
log2(FPKM+1)
0 1 2 3 4 5 6
0d 2d 4d 6d 14d Hep
C
Neuron projection morphogenesis
Muscle structure development
Heart development
Negative regulation of monocyte chemotaxis
Muscle cell proliferation
Supramolecular fiber organization
Regeneration
Regulation of neuron migration
Regulation of canonical Wnt signaling pathway
Positive regulation of interleukin-1 production
Trabecula formation
Regulation of synapse assembly
Central nervous system projection neuron axonogenesis
Negative regulation of neurological system process
Adherens junction organization
0.0 2.0 4.0 6.0 8.0
-log10(P-value)
D
cluster Gene expression
expressed 98
down-regulated 120
unexpressed 408
0d 2d 4d 6d 14d
log2(FPKM+1)
0 1 2
gene expression

Figure S5

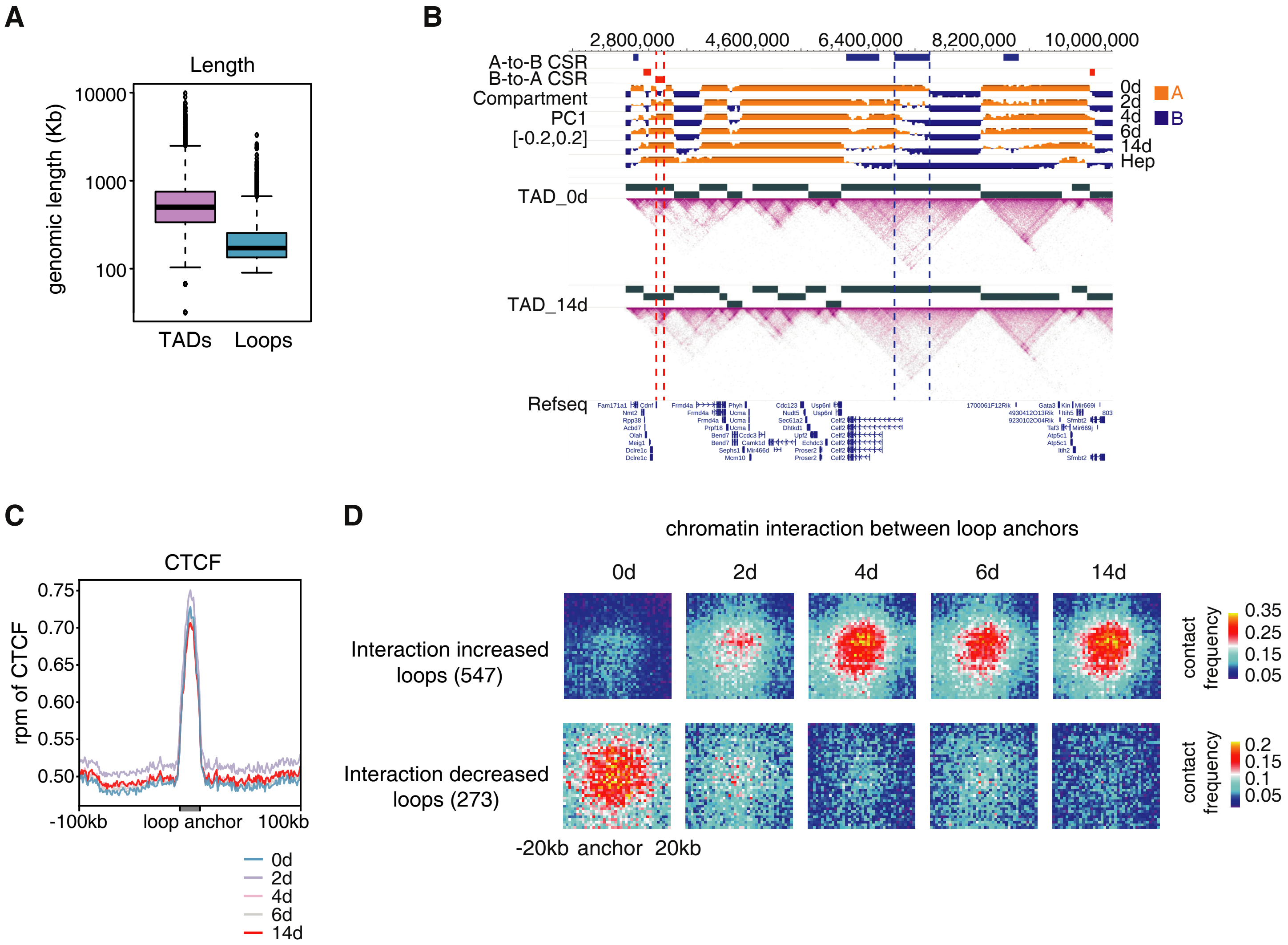
A
Length
10000
1000
100
genomic length (Kb)
TADs
Loops
B
2,800,000
4,600,000
6,400,000
8,200,000
10,000,000
A-to-B CSR
B-to-A CSR
Compartment
PC1
[-0.2,0.2]
0d
2d
4d
6d
14d
Hep
A
B
TAD_0d
TAD_14d
Refseq
C
CTCF
0.75
0.70
0.65
0.60
0.55
0.50
rpm of CTCF
-100kb
loop anchor
100kb
0d
2d
4d
6d
14d
D
chromatin interaction between loop anchors
0d
2d
4d
6d
14d
Interaction increased loops (547)
Interaction decreased loops (273)
contact frequency
0.35
0.25
0.15
0.05
0.2
0.15
0.1
0.05
-20kb
anchor
20kb

Figure S6

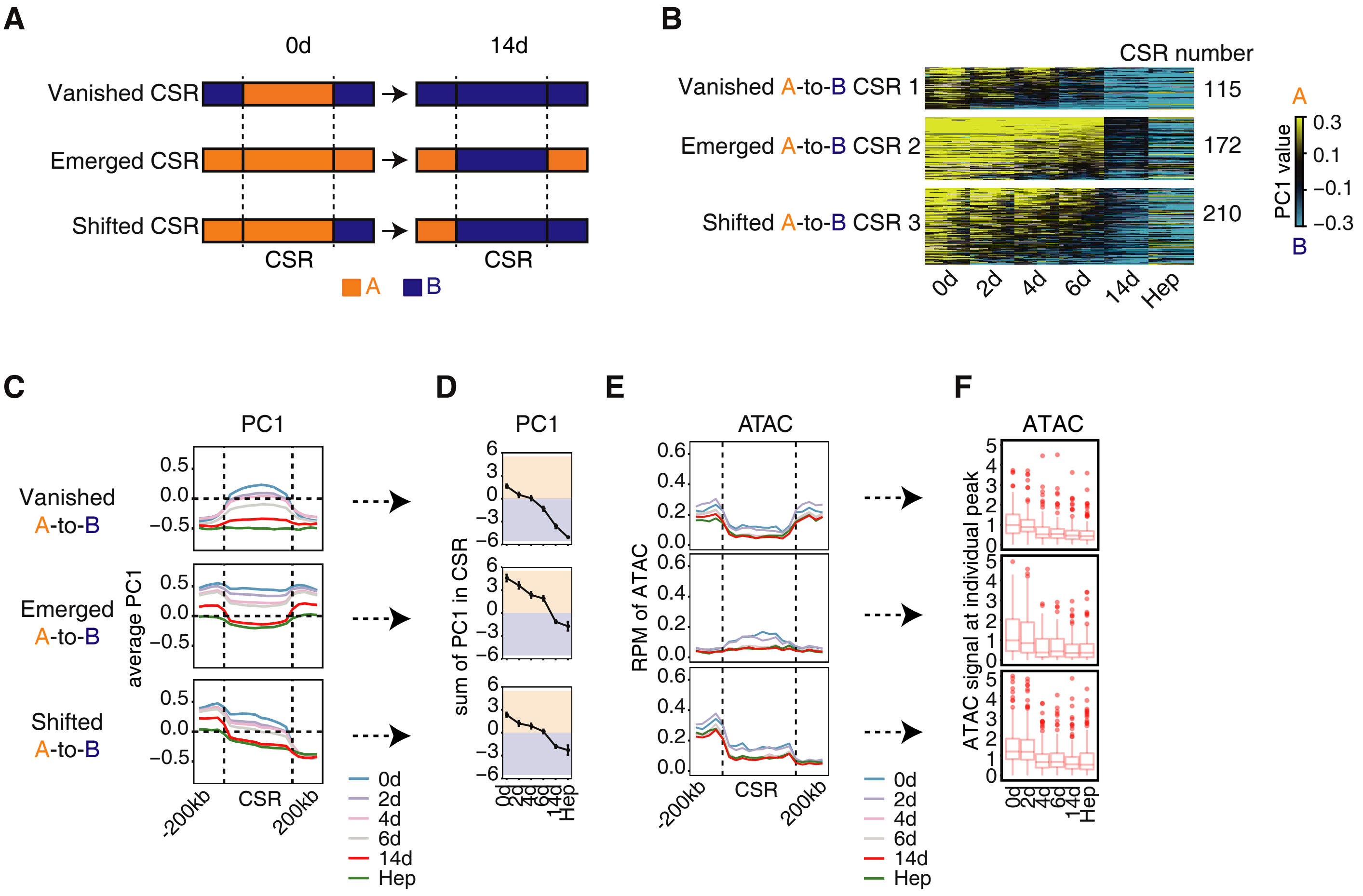
A
0d
14d
Vanished CSR
Emerged CSR
Shifted CSR
CSR
CSR
A
B
B
CSR number
Vanished A-to-B CSR 1
115
Emerged A-to-B CSR 2
172
Shifted A-to-B CSR 3
210
0d
2d
4d
6d
14d
Hep
A
0.3
0.1
−0.1
−0.3
PC1 value
B
C
PC1
Vanished A-to-B
Emerged A-to-B
Shifted A-to-B
average PC1
0.5
0.0
−0.5
-200kb
CSR
200kb
0d
2d
4d
6d
14d
Hep
D
PC1
sum of PC1 in CSR
6
3
0
−3
−6
E
ATAC
RPM of ATAC
0.6
0.4
0.2
0.0
-200kb
CSR
200kb
F
ATAC
ATAC signal at individual peak
5
4
3
2
1
0

Figure S7

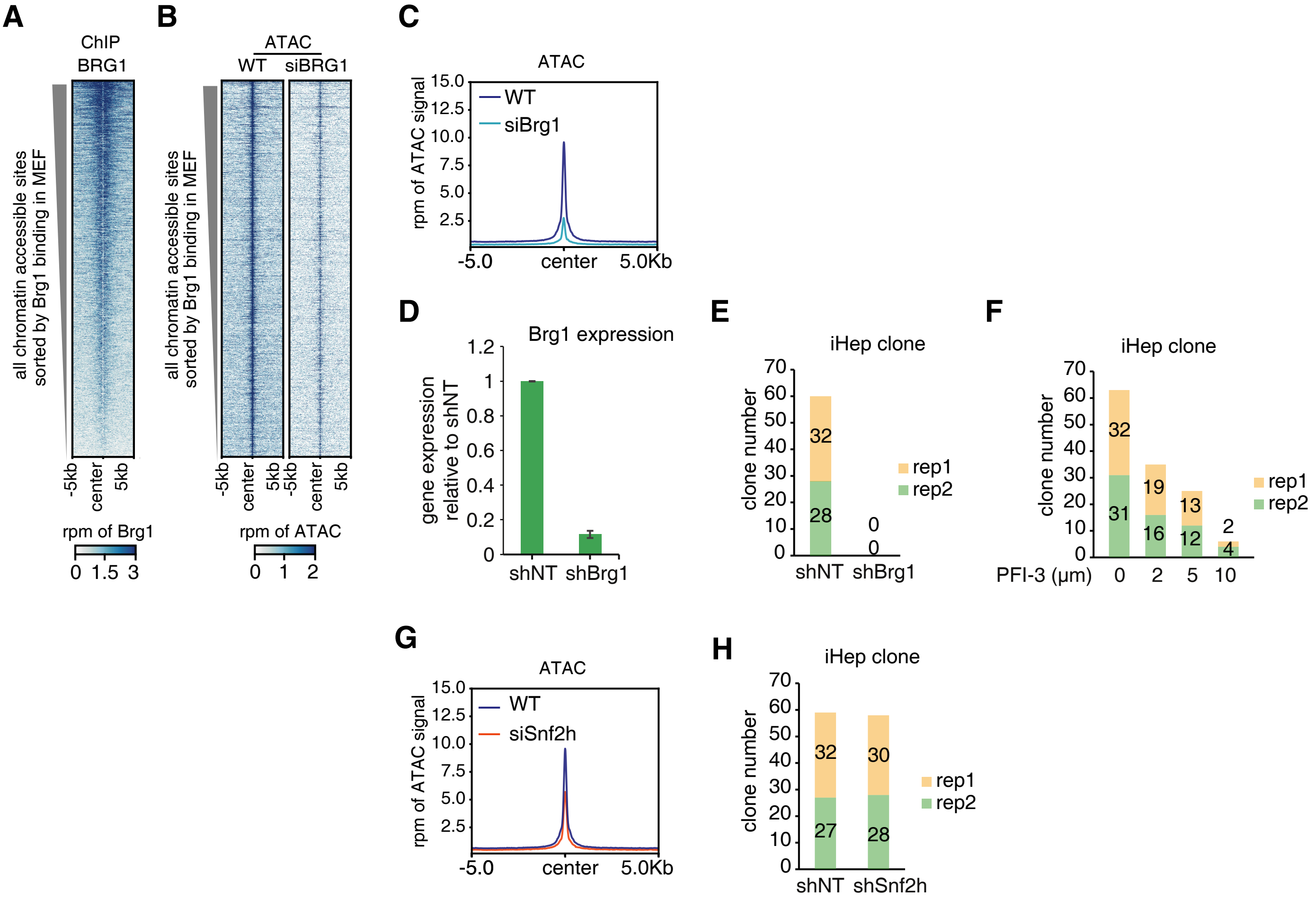
A
ChIP
BRG1
all chromatin accessible sites sorted by Brg1 binding in MEF
-5kb center 5kb
rpm of Brg1
0 1.5 3
B
ATAC
WT siBRG1
all chromatin accessible sites sorted by Brg1 binding in MEF
-5kb center 5kb -5kb center 5kb
rpm of ATAC
0 1 2
C
ATAC
WT
siBrg1
rpm of ATAC signal
15.0 12.5 10.0 7.5 5.0 2.5
-5.0 center 5.0Kb
D
Brg1 expression
gene expression relative to shNT
1.2 1 0.8 0.6 0.4 0.2 0
shNT shBrg1
E
iHep clone
clone number
70 60 50 40 30 20 10 0
32
28
0
0
rep1
rep2
shNT shBrg1
F
iHep clone
clone number
70 60 50 40 30 20 10 0
32
31
19
16
13
12
2
4
rep1
rep2
PFI-3 (μm) 0 2 5 10
G
ATAC
WT
siSnf2h
rpm of ATAC signal
15.0 12.5 10.0 7.5 5.0 2.5
-5.0 center 5.0Kb
H
iHep clone
clone number
70 60 50 40 30 20 10 0
32
27
30
28
rep1
rep2
shNT shSnf2h

Figure S8

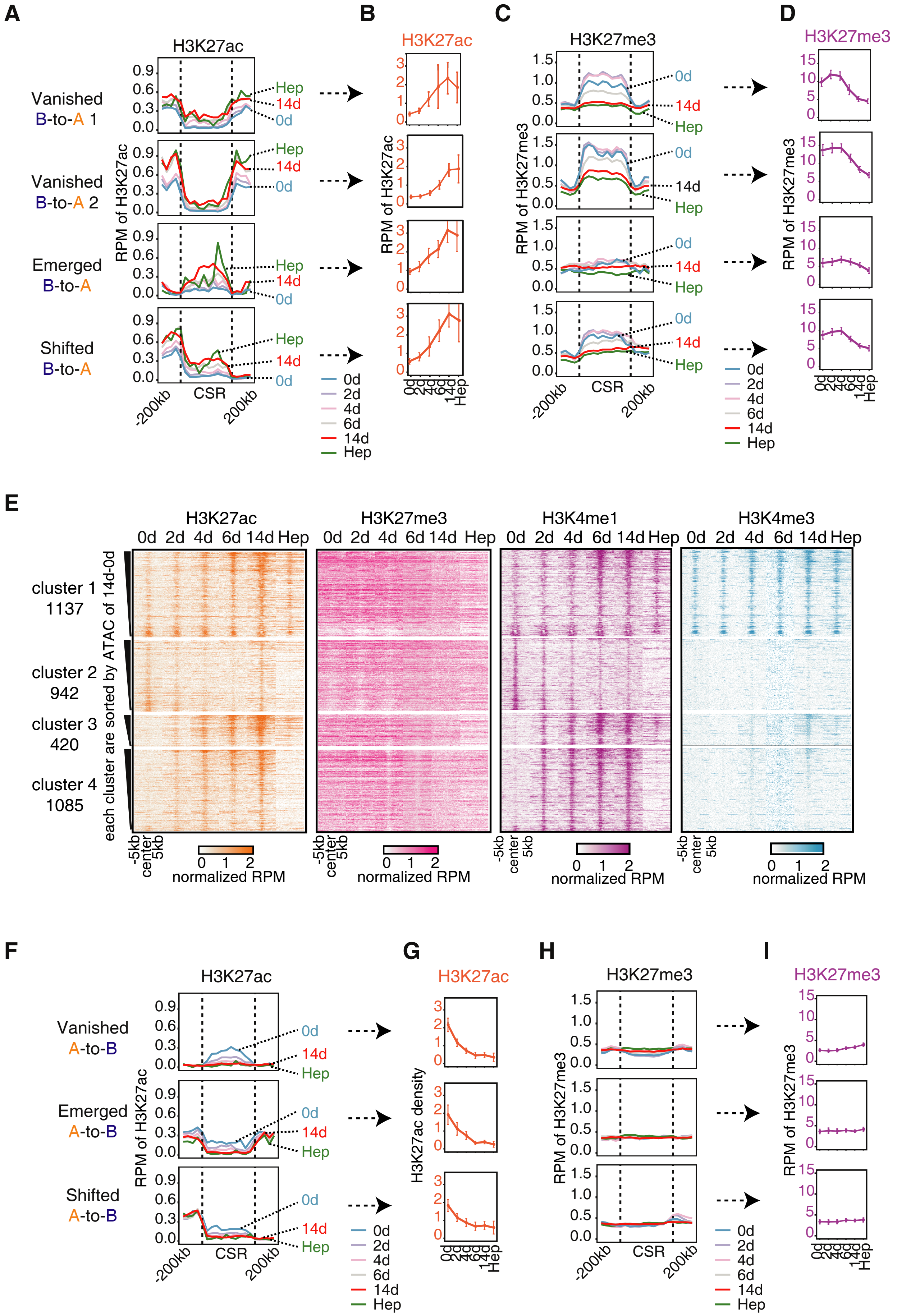
A
H3K27ac
Vanished B-to-A 1
Vanished B-to-A 2
Emerged B-to-A
Shifted B-to-A
RPM of H3K27ac
Hep
14d
0d
-200kb
CSR
200kb
B
H3K27ac
RPM of H3K27ac
C
H3K27me3
RPM of H3K27me3
D
H3K27me3
RPM of H3K27me3
0d
2d
4d
6d
14d
Hep
E
H3K27ac
H3K27me3
H3K4me1
H3K4me3
cluster 1 1137
cluster 2 942
cluster 3 420
cluster 4 1085
each cluster are sorted by ATAC of 14d-0d
-5kb center 5kb
normalized RPM
F
H3K27ac
Vanished A-to-B
Emerged A-to-B
Shifted A-to-B
RPM of H3K27ac
G
H3K27ac
H3K27ac density
H
H3K27me3
RPM of H3K27me3
I
H3K27me3
RPM of H3K27me3

Figure S9

A

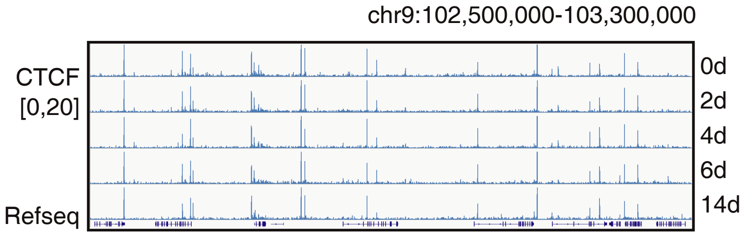
chr9:102,500,000-103,300,000
CTCF
[0,20]
Refseq
0d
2d
4d
6d
14d

B

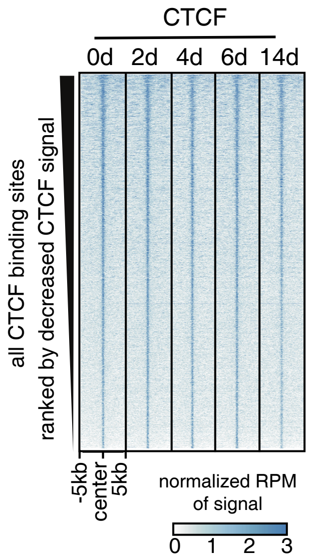
CTCF
0d
2d
4d
6d
14d
all CTCF binding sites
ranked by decreased CTCF signal
-5kb
center
5kb
normalized RPM
of signal
0
1
2
3

Figure S10

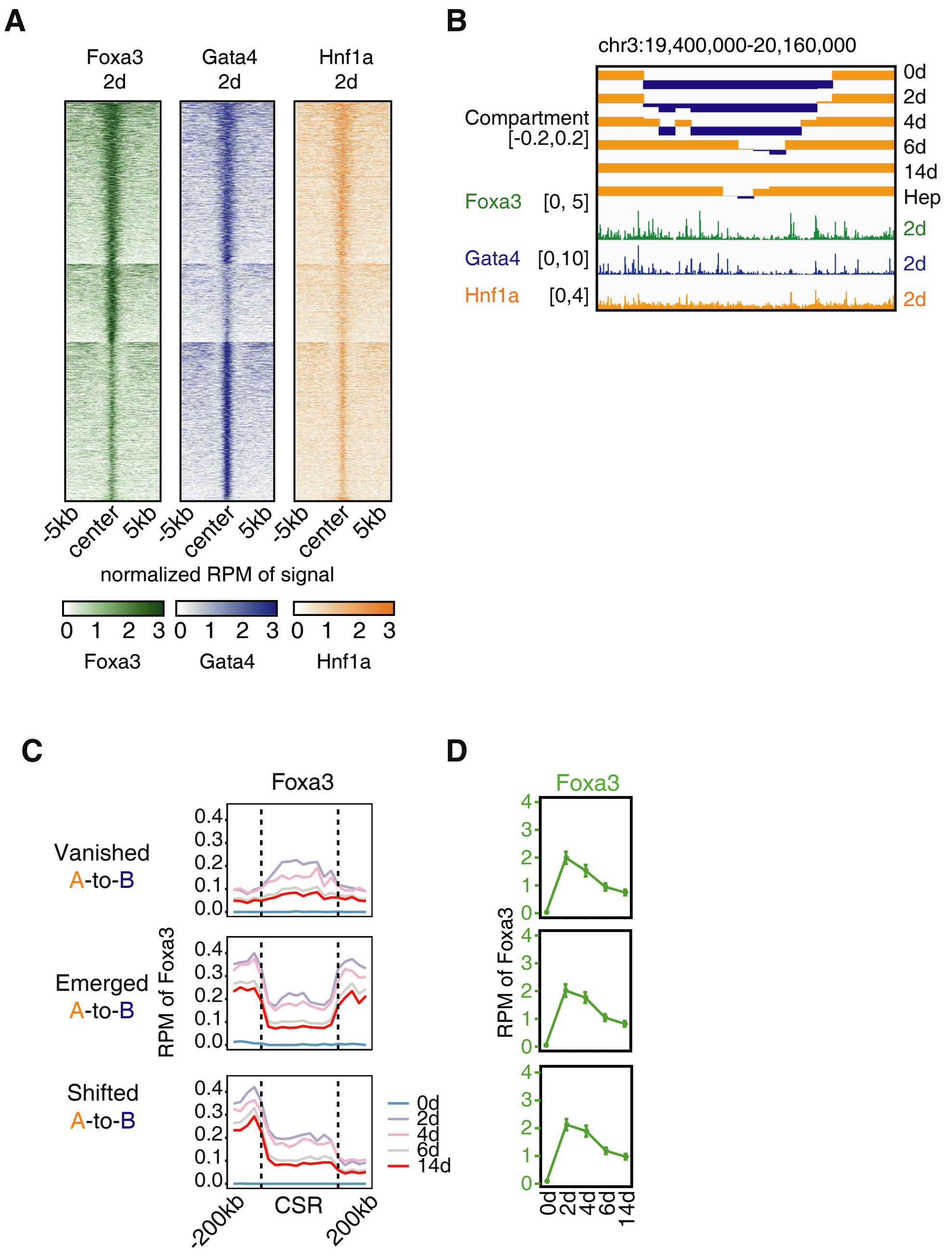
A
Foxa3 2d
Gata4 2d
Hnf1a 2d
-5kb
center
5kb
normalized RPM of signal
0 1 2 3
Foxa3
Gata4
Hnf1a
B
chr3:19,400,000-20,160,000
Compartment [-0.2,0.2]
0d
2d
4d
6d
14d
Hep
Foxa3 [0, 5]
Gata4 [0,10]
Hnf1a [0,4]
C
Foxa3
Vanished A-to-B
Emerged A-to-B
Shifted A-to-B
RPM of Foxa3
0.0
0.1
0.2
0.3
0.4
-200kb
CSR
200kb
0d
2d
4d
6d
14d
D
Foxa3
RPM of Foxa3
0
1
2
3
4
0d
2d
4d
6d
14d

Figure S11

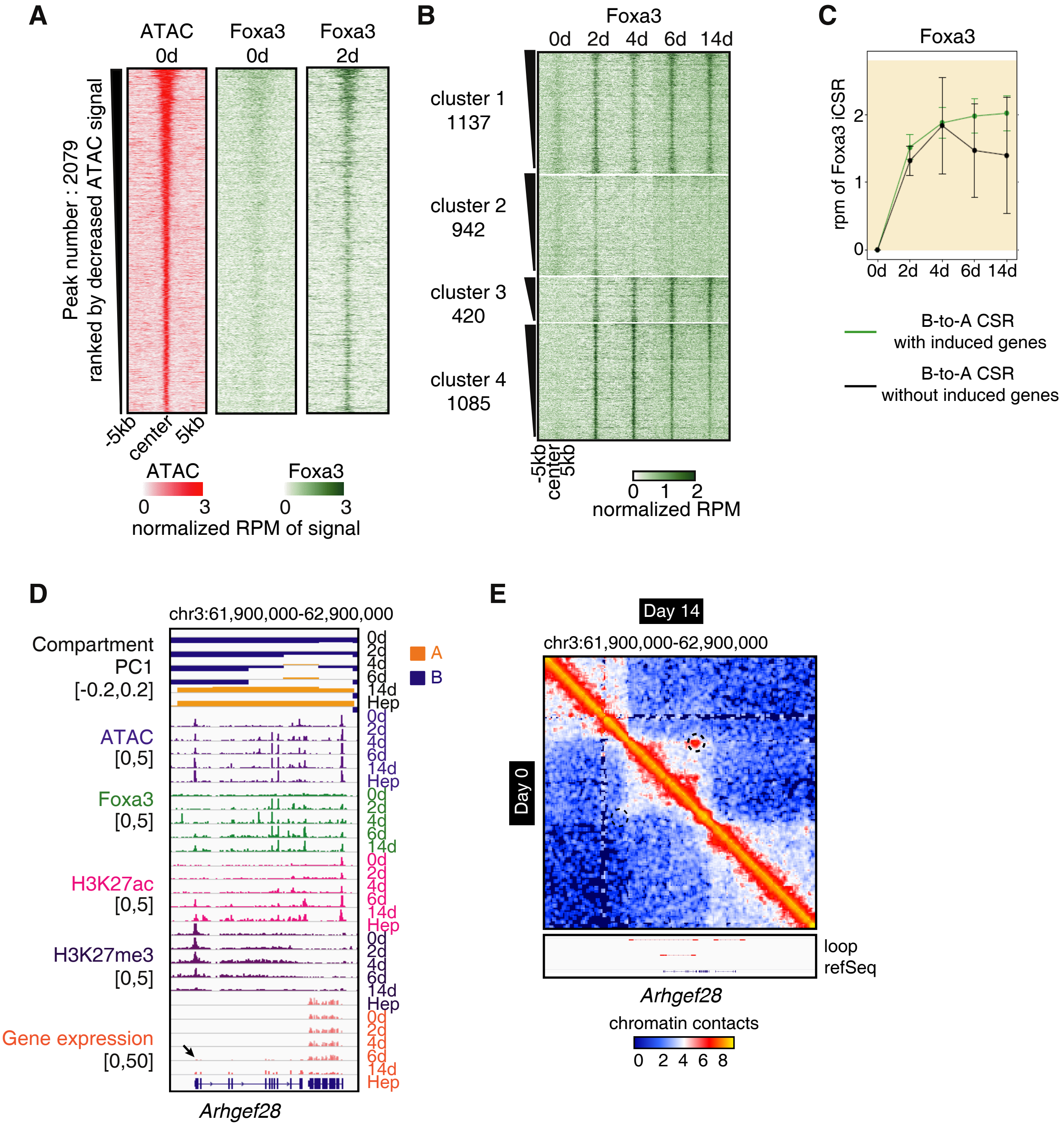

A
ATAC 0d
Foxa3 0d
Foxa3 2d
Peak number : 2079
ranked by decreased ATAC signal
-5kb
center
5kb
ATAC
0
3
Foxa3
0
3
normalized RPM of signal
B
Foxa3
0d 2d 4d 6d 14d
cluster 1
1137
cluster 2
942
cluster 3
420
cluster 4
1085
-5kb
center
5kb
0 1 2
normalized RPM
C
Foxa3
rpm of Foxa3 iCSR
0
1
2
0d 2d 4d 6d 14d
B-to-A CSR with induced genes
B-to-A CSR without induced genes
D
chr3:61,900,000-62,900,000
Compartment PC1 [-0.2,0.2]
0d 2d 4d 6d 14d Hep
A
B
ATAC [0,5]
0d 2d 4d 6d 14d Hep
Foxa3 [0,5]
0d 2d 4d 6d 14d
H3K27ac [0,5]
0d 2d 4d 6d 14d Hep
H3K27me3 [0,5]
0d 2d 4d 6d 14d Hep
Gene expression [0,50]
0d 2d 4d 6d 14d Hep
Arhgef28
E
Day 14
chr3:61,900,000-62,900,000
Day 0
loop
refSeq
Arhgef28
chromatin contacts
0 2 4 6 8

Figure S12

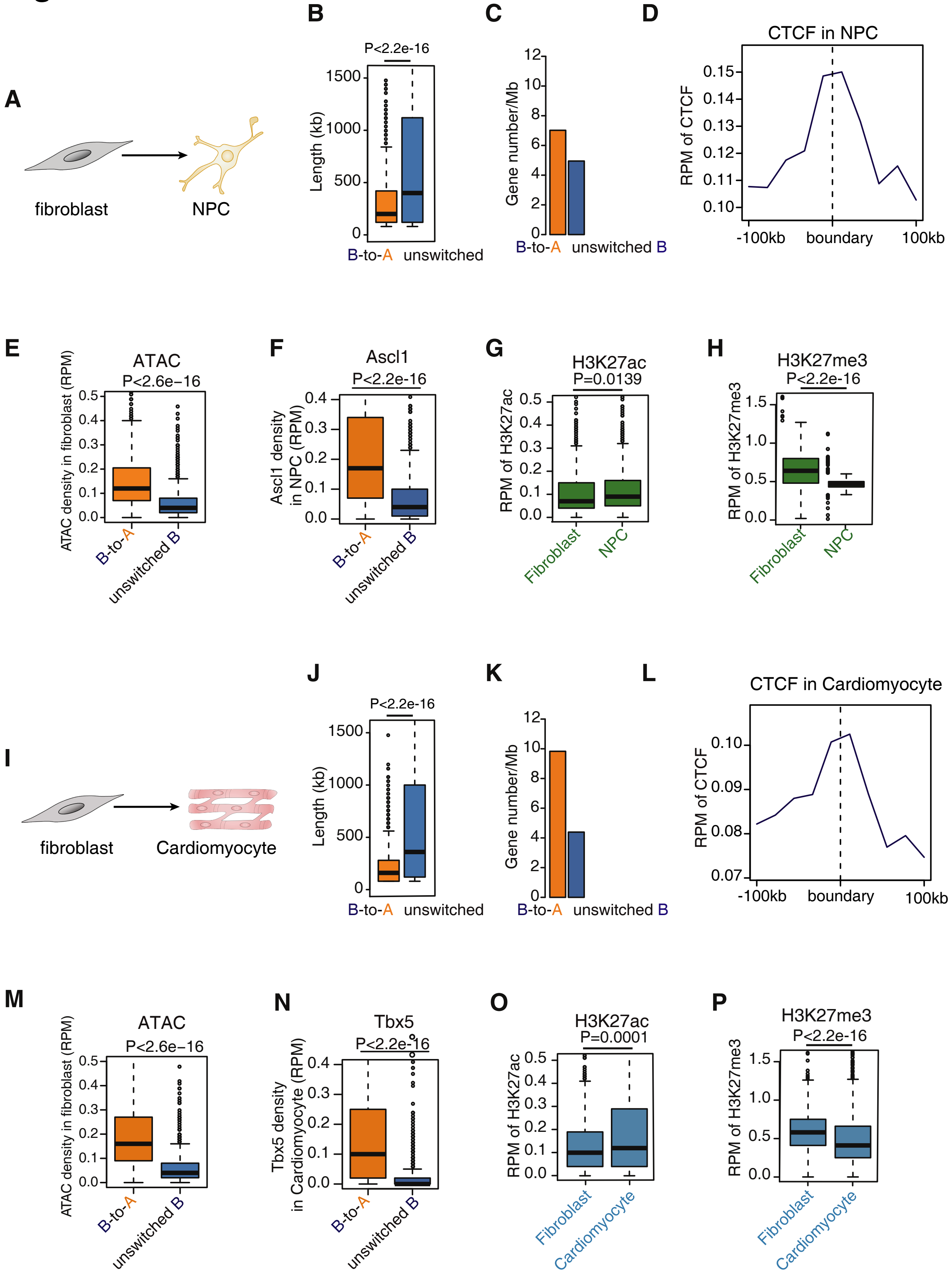
A
fibroblast
NPC
B
P<2.2e-16
Length (kb)
B-to-A
unswitched
C
Gene number/Mb
B-to-A
unswitched B
D
CTCF in NPC
RPM of CTCF
-100kb
boundary
100kb
E
ATAC
P<2.6e−16
ATAC density in fibroblast (RPM)
B-to-A
unswitched B
F
Ascl1
P<2.2e-16
Ascl1 density in NPC (RPM)
B-to-A
unswitched B
G
H3K27ac
P=0.0139
RPM of H3K27ac
Fibroblast
NPC
H
H3K27me3
P<2.2e-16
RPM of H3K27me3
Fibroblast
NPC
I
fibroblast
Cardiomyocyte
J
P<2.2e-16
Length (kb)
B-to-A
unswitched
K
Gene number/Mb
B-to-A
unswitched B
L
CTCF in Cardiomyocyte
RPM of CTCF
-100kb
boundary
100kb
M
ATAC
P<2.6e−16
ATAC density in fibroblast (RPM)
B-to-A
unswitched B
N
Tbx5
P<2.2e-16
Tbx5 density in Cardiomyocyte (RPM)
B-to-A
unswitched B
O
H3K27ac
P=0.0001
RPM of H3K27ac
Fibroblast
Cardiomyocyte
P
H3K27me3
P<2.2e-16
RPM of H3K27me3
Fibroblast
Cardiomyocyte

Figure S13

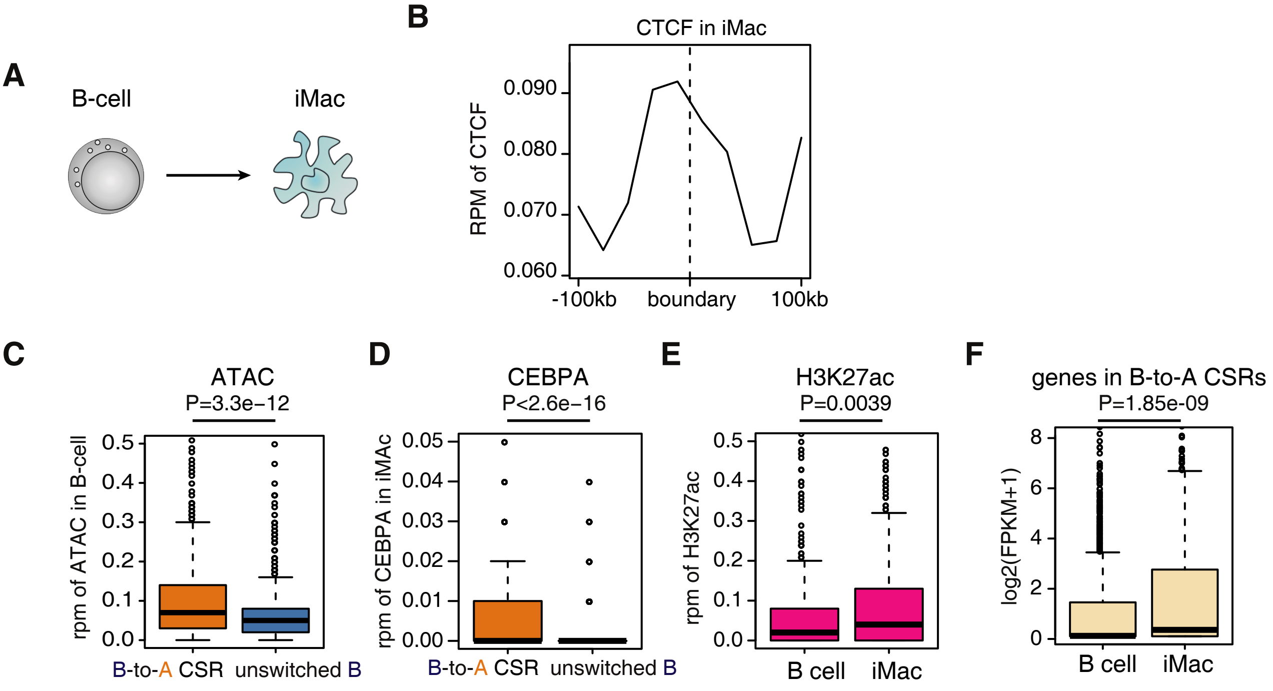

A
B-cell
iMac
B
CTCF in iMac
RPM of CTCF
0.090
0.080
0.070
0.060
-100kb
boundary
100kb
C
ATAC
P=3.3e−12
rpm of ATAC in B-cell
0.5
0.4
0.3
0.2
0.1
0.0
B-to-A CSR
unswitched B
D
CEBPA
P<2.6e−16
rpm of CEBPA in iMAc
0.05
0.04
0.03
0.02
0.01
0.00
B-to-A CSR
unswitched B
E
H3K27ac
P=0.0039
rpm of H3K27ac
0.5
0.4
0.3
0.2
0.1
0.0
B cell
iMac
F
genes in B-to-A CSRs
P=1.85e-09
log2(FPKM+1)
8
6
4
2
0
B cell
iMac
